# Research on prognostic prediction of colorectal cancer based on multi-dimensional biomarker features and machine learning models

**DOI:** 10.3389/fonc.2026.1855784

**Published:** 2026-09-04

**Authors:** Xiaoyang Zhang, Xiangyong Li, Bo Chen, Xinmeng Cheng, Yuee He, Chenxi Zhou, Jinquan Liu, Xiaodong Yang

**Affiliations:** 1 Department of Gastrointestinal Surgery, The Second Affiliated Hospital of Soochow University, Suzhou, Jiangsu, China; 2 Nursing Department, The Second Affiliated Hospital of Soochow University, Suzhou, China

**Keywords:** colorectal cancer, machine learning, multi-dimensional biomarkers, precision medicine, prognostic prediction

## Abstract

**Background:**

The traditional TNM staging system for colorectal cancer (CRC) prognosis has significant limitations, with prognostic differences exceeding 40% among patients with the same stage. This study aims to construct and validate a machine learning prognostic prediction model based on routinely available clinical, laboratory, inflammatory, nutritional, and treatment-related variables for hospitalized colorectal cancer patients. Given that the training cohort (MIMIC-IV) primarily comprises hospitalized and critically ill patients, the proposed model is intended for risk stratification in hospitalized CRC patients.

**Methods:**

A retrospective cohort study used the MIMIC-IV database (n = 2049) for training/internal validation and the Second Affiliated Hospital of Soochow University (n = 561) for external validation. The study integrated characteristics including clinical pathology, inflammatory response, immune function, nutritional status, tumor markers, and treatment information. Through correlation analysis, collinearity testing, and the Boruta algorithm, 16 core features were selected. Nine machine learning algorithms were employed to construct the model, with hyperparameters optimized via five-fold cross-validation and grid search. Model performance was evaluated using AUROC, AUPR, Brier score, survival consistency index, and decision curve analysis, with feature contribution explained by SHAP values.

**Results:**

The RSF model achieved the highest normalized comprehensive score among the nine algorithms evaluated. In the external validation set, the RSF model achieved an average AUROC of 0.818 (95% CI: 0.761-0.866), ranking first in this composite metric. The full RSF model showed statistically significantly higher AUROC than TNM-only baseline models (DeLong test P < 0.001), suggesting potential for incremental prognostic information beyond traditional staging, pending prospective validation. Feature importance analysis revealed that age-corrected Charlson Comorbidity Index (ACCI), distant metastasis stage (Mstage), and surgical treatment were core prognostic factors, exhibiting time-dependent dynamic changes. Inflammatory markers (CRP), nutritional markers (ALB, TC), and tumor markers (CEA) also demonstrated significant predictive value.

**Conclusions:**

The RSF model demonstrated favorable discrimination and calibration in the evaluated cohorts; clinical utility requires prospective evaluation in the available cohorts, showing incremental prognostic value beyond TNM staging alone. These findings suggest potential for assisting risk stratification in similar hospitalized populations, although further prospective and multicenter validation remains necessary before clinical implementation.

## 1 Introduction

Colorectal cancer (CRC) is the third most common cancer globally and the second leading cause of cancer-related deaths (1, 2). China’s CRC incidence ranks second among malignant tumors, with a mortality rate in fourth place, and the proportion of young patients has significantly increased (3, 4). Despite advances in treatment technologies, the 5-year survival rate for stage IV patients is only about 31.5%, and approximately 20–25% of patients have distant metastases at diagnosis (5–7). Therefore, developing refined prognostic assessment tools may assist in informing personalized treatment decisions.

Current clinical practice primarily relies on the AJCC-TNM staging system, which fails to adequately account for prognostic heterogeneity at the clinical and laboratory level, as patients with the same staging exhibit significant differences in recurrence risk and long-term survival (8, 9). Traditional serum markers such as carcinoembryonic antigen (CEA) demonstrate limited sensitivity and specificity (10). In recent years, the integration of multidimensional biomarkers has become a research focus, encompassing circulating tumor DNA (ctDNA) methylation, programmed cell death (PCD) pathways, inflammation-nutrition indicators, and radiomics (10–12). Recent studies have further highlighted the prognostic value of inflammation-nutrition biomarkers in CRC. The platelet-albumin-based Cancer Inflammation Prognostic Index (CIPI) has been identified as an independent predictor of recurrence and survival in nonmetastatic CRC (13). Hematologic inflammatory markers, including NLR, PLR, and LMR, demonstrate consistent associations with CRC prognosis across diverse clinical settings (14). Serum nutritional biomarkers such as albumin and total cholesterol reflect host metabolic reserve and have been linked to postoperative outcomes and long-term survival in CRC patients (15). Machine learning algorithms, leveraging their advantages in processing high-dimensional data and nonlinear relationships, have demonstrated potential in medical prognostic prediction (16, 17). However, existing studies are often limited to single omics data and lack independent external validation across diverse clinical settings.

The innovative aspects of this study include (1): Data integration: Comprehensive capture of prognostic information by combining routinely available clinical pathology, inflammation, immunity, nutrition, treatment data, and composite indicators (e.g., Systemic Immune-Inflammation Index [SII], Glasgow Prognostic Score [GPS]) from electronic health records; it should be noted that molecular pathology data (e.g., MSI, KRAS, BRAF, ctDNA) were not included in this study (2); Model optimization: Systematic comparison of nine machine learning algorithms to establish the optimal model through rigorous validation strategies (3); Clinical translation: Preliminary steps toward clinical translation include independent external validation and development of a Shiny web application for exploratory use.

## 2 Materials and methods

### 2.1 Data sources and study subjects

**Figure 1:**
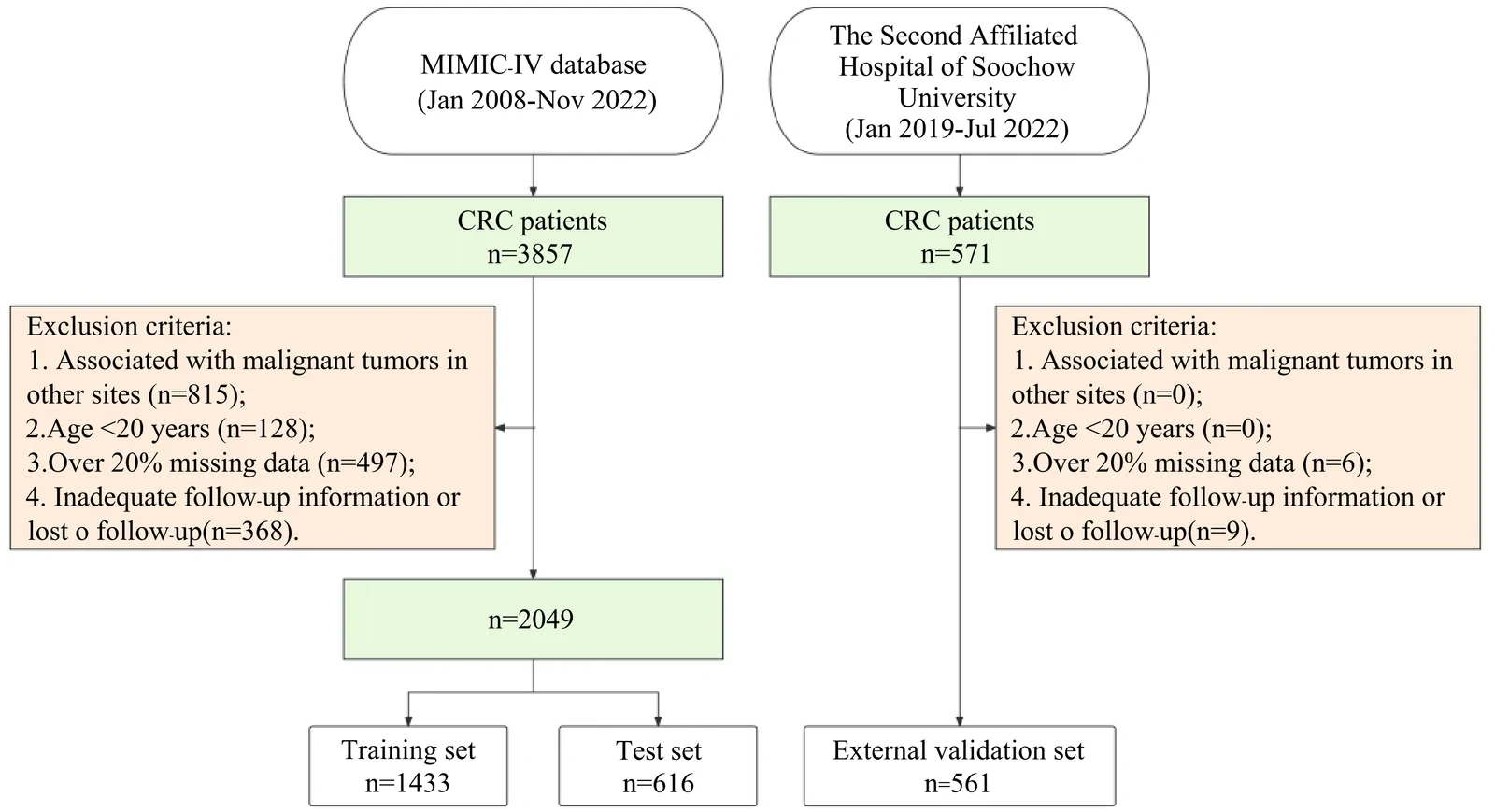
Flowchart of patient selection and exclusion process.

This study is a retrospective cohort study conducted in accordance with the TRIPOD protocol and approved by the Ethics Committee of the Second Affiliated Hospital of Soochow University (SUFAH), Approval No.: JD-HG-2025-125 (18). The training set and internal validation set were sourced from the MIMIC-IV- 3.1 electronic health record (EHR) database (covering admissions from 2008 to 2022) (19), while the external validation set was obtained from the single-center SUFAH cohort (patient admissions from January 2019 to July 2022). The patient screening time window and cohort period are consistently presented in **Figure 1** and **Table 1**.

**Table 1:** Patient characteristics of the MIMIC database and the SUFAH.

Characteristic	MIMIC cohort (n=2049)	SUFAH cohort (n=561)	*P*
Survival status			0.001
Survival	1223 (59.7%)	379 (67.6%)	
Death	826 (40.3%)	182 (32.4%)	
Overall Survival (days)	1980 (1737)	1615 (602)	<0.001
Age (years)	66.7 (14.9)	65.6 (12.4)	0.086
BMI (kg/m^2^)	27.7 (9.70)	23.2 (3.25)	<0.001
CRP (mg/L)	50.1 (59.5)	18.1 (35.8)	<0.001
LYM (×10^9^/L)	1.31 (2.54)	1.41 (0.76)	0.154
PLA (×10^9^/L)	261 (115)	240 (87.4)	<0.001
NEUT (×10^9^/L)	7.19 (4.03)	4.05 (2.28)	<0.001
MON (×10^9^/L)	0.68 (0.49)	0.66 (0.77)	0.637
WBC (×10^9^/L)	9.49 (4.93)	6.12 (2.55)	<0.001
TP (g/dL)	5.84 (0.96)	6.69 (0.69)	<0.001
ALB (g/dL)	3.16 (0.64)	4.00 (0.50)	<0.001
HGB (g/dL)	11.7 (1.79)	11.9 (2.69)	0.018
TC (mg/dL)	140 (49.1)	437 (100)	<0.001
FIB (mg/dL)	405 (173)	373 (89.6)	<0.001
CEA (ng/mL)	45.9 (27.26)	14.7 (34.4)	<0.001
NLR	9.12 (10.8)	3.82 (4.28)	<0.001
PLR	324 (344)	207 (138)	<0.001
LMR	2.82 (4.40)	2.54 (1.38)	0.021
PIV	1712 (3013)	621 (1135)	<0.001
PNI	31.6 (6.48)	40.0 (4.99)	<0.001
SII	2532 (4132)	926 (1103)	<0.001
Gender			0.024
Male	1075 (52.5%)	325 (57.9%)	
Female	974 (47.5%)	236 (42.1%)	
Race			0
White	1462 (71.4%)	0 (0.00%)	
Black	252 (12.3%)	0 (0.00%)	
Yellow	142 (6.93%)	561 (100%)	
Other	97 (4.73%)	0 (0.00%)	
Unknown	96 (4.69%)	0 (0.00%)	
ACCI			<0.001
0 (<5)	467 (22.8%)	402 (71.7%)	
1 (5-7)	764 (37.3%)	108 (19.3%)	
2 (>=8)	818 (39.9%)	51 (9.09%)	
T stage			<0.001
T1	543 (26.5%)	47 (8.38%)	
T2	452 (22.1%)	97 (17.3%)	
T3	586 (28.6%)	385 (68.6%)	
T4	468 (22.8%)	32 (5.70%)	
N stage			<0.001
N0	1131 (55.2%)	322 (57.4%)	
N1	466 (22.7%)	177 (31.6%)	
N2	452 (22.1%)	62 (11.1%)	
M stage			<0.001
M0	1370 (66.9%)	533 (95.0%)	
M1	679 (33.1%)	28 (4.99%)	
Surgery			<0.001
No	858 (41.9%)	21 (3.74%)	
Yes	1191 (58.1%)	540 (96.3%)	
Radiotherapy			0.394
No	1793 (87.5%)	499 (88.9%)	
Yes	256 (12.5%)	62 (11.1%)	
Chemotherapy			0.991
No	1723 (84.1%)	471 (84.0%)	
Yes	326 (15.9%)	90 (16.0%)	
NPS			<0.001
Low (0)	8 (0.39%)	1 (0.18%)	
Medium (1)	156 (7.61%)	71 (12.7%)	
High (2)	1011 (49.3%)	489 (87.2%)	
NA	874 (42.7%)	0 (0.00%)	
GPS			<0.001
Low (0)	245 (12.0%)	389 (69.3%)	
Medium (1)	623 (30.4%)	120 (21.4%)	
High (2)	753 (36.7%)	52 (9.27%)	
NA	428 (20.9%)	0 (0.00%)	

SUFAH, The Second Affiliated Hospital of Soochow University; BMI, Body Mass Index; CRP, C-Reactive Protein; LYM, Lymphocyte Count; PLA, Platelet Count; NEUT, Neutrophil Count; MON, Monocyte Count; WBC, White Blood Cell Count; TP, Total Protein; ALB, Albumin; HGB, Hemoglobin; TC, Total Cholesterol; FIB, Fibrinogen; CEA, Carcinoembryonic Antigen; NLR, Neutrophil-to-Lymphocyte Ratio; PLR, Platelet-to-Lymphocyte Ratio; LMR, Lymphocyte-to-Monocyte Ratio; PIV, Pan-Immune-Inflammation Value; PNI, Prognostic Nutritional Index; SII, Systemic Immune-Inflammation Index; ACCI, Age-Adjusted Charlson Comorbidity Index; TNM, Tumor Node Metastasis; NPS, Naples Prognostic Score; GPS, Glasgow Prognostic Score.SUFAH TC originally measured in mmol/L, converted to mg/dL using 38.67.

Inclusion criteria (1): CRC diagnosis identified by ICD-9 codes (153.X, 154.X) or ICD-10 codes (C18.X-C21.X) in the MIMIC-IV database, and pathologically confirmed CRC in the SUFAH cohort (2); Age ≥20 years. In the MIMIC-IV database, CRC cases were identified through ICD diagnostic codes and subsequently cross-validated with pathology reports where available. Among 2,049 included cases, pathology reports were accessible for 1,247 (60.9%) patients; ICD-pathology concordance was 94.2% (1,175/1,247). The remaining 802 cases without accessible pathology reports were retained based on multiple ICD code occurrences (≥2 separate encounters with CRC diagnosis codes) to reduce false-positive inclusion (**Supplementary Table 2**). In the SUFAH cohort, all cases were confirmed by histopathological examination of surgical specimens or biopsy samples. Exclusion criteria: Concurrent other malignancies; Missing values >20%; Incomplete follow-up data. The outcome event was all-cause mortality, with follow-up ending on April 30, 2025. The patient screening method is shown in **Figure 1**.

### 2.2 Data collection and variable definition

#### 2.2.1 Characteristic variables

Including Age, Gender, Race, body mass index (BMI), age-adjusted Charlson Comorbidity Index(ACCI), TNM staging, inflammatory markers, nutritional markers, tumor markers, treatment modalities, and composite indicators. TNM staging: In the MIMIC-IV database, TNM stage was extracted from structured clinical data fields and cross-referenced with discharge summaries and pathology reports. In the SUFAH cohort, TNM stage was determined by the multidisciplinary tumor board based on pathological examination, imaging studies (CT/MRI), and intraoperative findings, classified according to the AJCC 8th edition. Treatment information: Surgery was defined as any curative or palliative resection of the primary tumor or metastatic lesions performed during the index hospitalization. Radiotherapy and chemotherapy were coded as binary variables (Yes/No) based on treatment records during the index admission. In the SUFAH cohort, treatment information was obtained from electronic medical records and verified against the hospital’s oncology registry. Laboratory indicators were defined as the first available measurement within 48 hours of hospital admission (baseline window), prior to any cancer-directed treatment (surgery, chemotherapy, or radiotherapy). If multiple tests were performed within this window, the value closest to admission was used. This approach was adopted to minimize information bias and ensure that laboratory values reflected the patient’s pre-treatment baseline status rather than post-treatment or complication-related changes. For patients who underwent emergency surgery within 48 hours of admission, preoperative laboratory values were used when available. All variables and data sources are detailed in **Supplementary Table 1**.

#### 2.2.2 ACCI calculation

The sum of the comorbidity score (myocardial infarction, heart failure, etc., scored 1–6 points) and the age score (≥50 years, 1 point per 10 years), with <5 points scored as 0, 5–7 points scored as 1, and ≥8 points scored as 2.

#### 2.2.3 Data preprocessing

Categorical variables with unordered categories were encoded using one-hot encoding, while ordered categorical variables (e.g., ACCI) were encoded using label encoding. Continuous variables were standardized using Z-score, with parameters calculated solely based on the training set and applied to the test and validation sets. Missing values were handled using multiple imputation (MI) combined with five-fold cross-validation (20). Feature selection was performed through correlation analysis (Pearson r > 0.6), collinearity testing (VIF > 5 deletion), and the Boruta algorithm (21).

### 2.3 Model construction and validation

**Figure 2:**
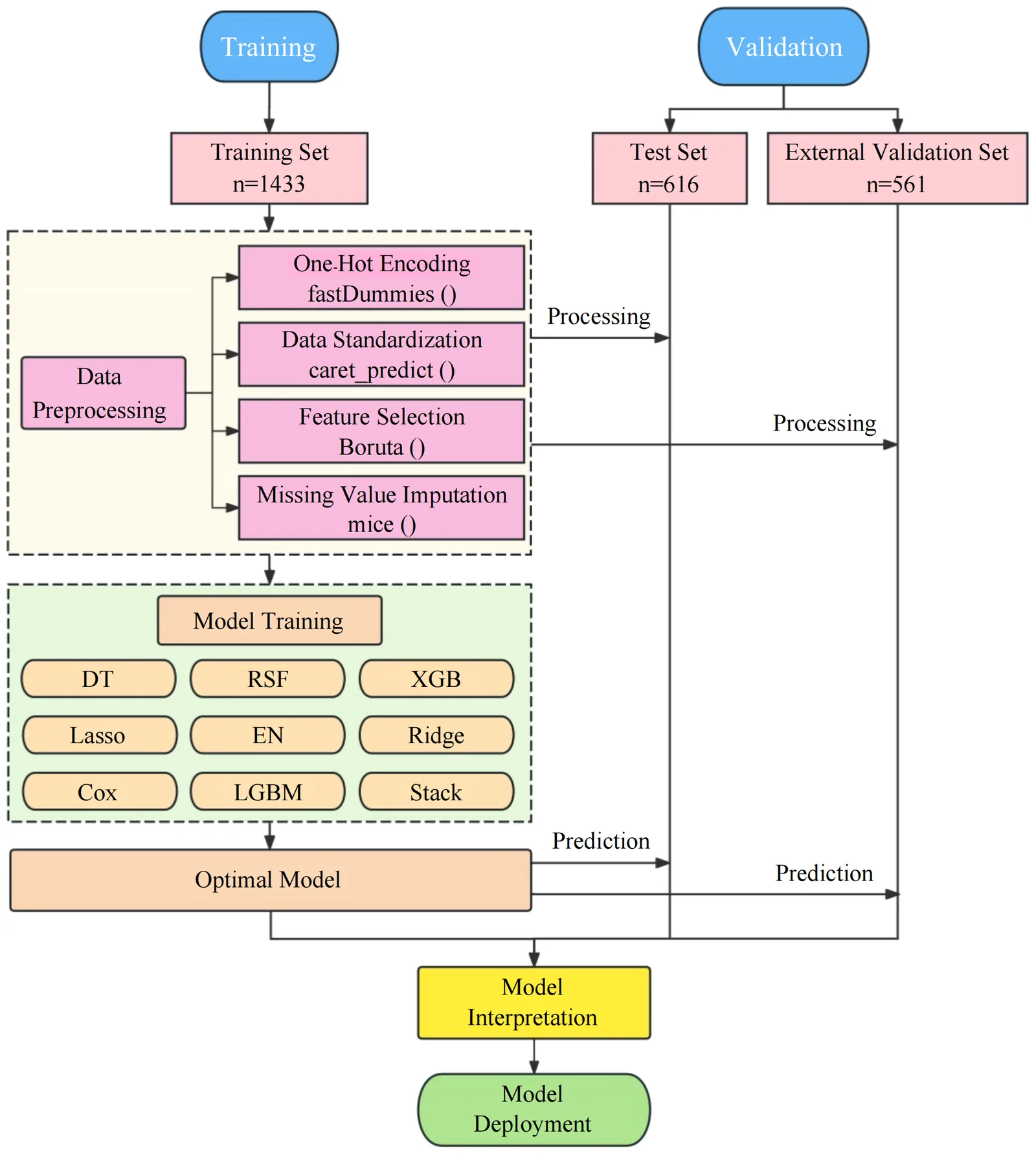
Overview of the study design.

The ‘three-way partition method’ was adopted: MIMIC data was divided into a training set and an internal validation set in a 7:3 ratio using stratified random sampling, while the data from the SUFAH served as the external validation set. The external validation set was used exclusively for final model assessment and was not involved in model selection, hyperparameter tuning, or feature selection. Model selection was based on internal validation performance (test set) and the predefined comprehensive scoring metric (**Figure 2**).

#### 2.3.1 Algorithm selection

Decision Tree (DT), Random Survival Forest (RSF), Extreme Gradient Boosting (XGB), Lasso regression, Elastic Net (EN), Ridge Regression, Cox Proportional Hazards Model (Cox), Light Gradient Boosting Machine (LGBM), and Stack ensemble algorithm. Hyperparameter optimization employed grid search combined with five-fold cross-validation, with the survival consistency index (surv.cindex) as the evaluation metric.

#### 2.3.2 Model evaluation metrics

The Area Under the Receiver Operating Characteristic (AUROC), Area Under the Precision-Recall Curve (AUPR), and surv.cindex (95% CI calculated via 200 bootstrap iterations) were used to assess model discrimination ability; Brier score and calibration curve were employed to evaluate calibration; decision curve analysis (DCA) was utilized to assess clinical net benefit (22, 23). The comprehensive score was calculated as the mean of AUROC, AUPR, and negative Brier score after min-max normalization to a 0–1 scale.

#### 2.3.3 Model interpretation

SHapley Additive exPlanations (SHAP) was employed to analyze the temporal dependence of feature contributions, including feature importance ranking, partial dependence graphs (PDPs), and individual-level SHAP force-directed graphs (24). It is important to note that SHAP and PDP analyses explain model predictions but do not establish causal relationships or therapeutic benefit.

#### 2.3.4 Model deployment

The Shiny web application was developed using the optimal RSF model and deployed on the shinyapps.io platform. It is an exploratory decision-support tool rather than a validated clinical platform.

#### 2.3.5 Stepwise incremental-value analysis

To systematically evaluate the incremental prognostic contribution of distinct feature domains, we constructed a hierarchical sequence of nested models using the RSF algorithm with consistent hyperparameter search protocols. The model hierarchy was prespecified as follows (1): Model 0 (TNM-only): Tstage, Nstage, Mstage, representing the conventional AJCC 8th edition staging (2); Model 1 (TNM + Demographics/Comorbidity): Model 0 variables plus Age and ACCI, quantifying baseline health status (3); Model 2 (TNM + Treatment): Model 0 variables plus Surgery, Chemotherapy, and Radiotherapy, assessing treatment modality contributions (4); Model 3 (TNM + Laboratory): Model 0 variables plus CRP, ALB, CEA, TC, TP, HGB, and WBC, evaluating inflammatory, nutritional, and tumor marker dimensions (5); Model 4 (Full RSF): All 16 confirmed features, the final integrated model. Each successive model was compared with its immediate predecessor using the DeLong test for AUROC differences and bootstrap resampling (200 iterations) for surv.cindex and Brier score differences. The incremental contribution of each feature domain was quantified by ΔAUROC, ΔAUPR, Δsurv.cindex, and ΔBrier score. To ensure fair comparison, all models were trained on identical data partitions with the same random seeds and evaluated on the held-out external validation set (SUFAH cohort).

#### 2.3.6 Sensitivity analysis

To assess the potential impact of treatment-related variables on model performance, we conducted a sensitivity analysis excluding Surgery, Chemotherapy, and Radiotherapy from the feature set. The RSF model without treatment variables achieved an average external validation AUROC of 0.814 (95% CI: 0.762-0.846), and surv.cindex of 0.772 (95% CI: 0.730-0.804). While performance was modestly reduced compared to the full model (AUROC difference: 0.004), the model retained good discriminative ability, suggesting that the prognostic value of the multidimensional feature set is not solely dependent on treatment variables (**Supplementary Figure 1**).

#### 2.3.7 Landmark analysis

To further assess potential treatment variable leakage, we conducted a landmark analysis censoring patients at 7 days post-admission and re-evaluating Model 4 using only pre-landmark treatment information. Of 561 SUFAH patients, 23 (4.1%) experienced death within 7 days and were excluded from landmark analysis, leaving 538 patients. Baseline characteristics of landmark subset were comparable to full cohort. The landmark AUROC at t=365 days was 0.809 (vs. 0.812 in primary analysis, Δ−0.003), confirming minimal leakage impact (**Supplementary Figure 1**).

### 2.4 Statistical analysis

Baseline characteristics comparison: Continuous variables were analyzed using Student t-test, and categorical variables were analyzed using Pearson chi-square test. Survival analysis was performed using Kaplan-Meier method and Log-rank test. All analyses were conducted using R software (v4.5.1), with P < 0.05 considered statistically significant.

## 3 Results

### 3.1 Patient characteristics

This study screened 2,049 and 561 eligible CRC patients from the MIMIC-IV database and the SUFAH, respectively, for analysis. The results demonstrated that the two cohorts exhibited demographic similarities, such as average age (MIMIC: 66.7 ± 14.9 years; SUFAH: 65.6 ± 12.4 years, P = 0.086) and male proportion (MIMIC: 52.5%; SUFAH: 57.9%, P = 0.024). However, significant statistical differences were observed between the two datasets in multiple other characteristics (P < 0.05). Specifically, the mortality rate (40.3% vs. 32.4%, P = 0.001) and average overall survival (1,980 ± 1,737 days vs. 1,615 ± 602 days, P < 0.001) in the MIMIC dataset differed from those in the SUFAH cohort. The MIMIC dataset exhibited a higher burden of comorbidities among patients, such as a greater proportion of ACCI = 2 (39.9% vs. 9.09%, P<0.001), higher BMI (27.7 ± 9.70 vs. 23.2 ± 3.25, P<0.001), and more advanced tumor progression, as evidenced by significantly higher proportions of T4-stage tumors (22.8% vs. 5.70%, P<0.001), N2 lymph node metastases (22.1% vs. 11.1%, P<0.001), and M1 distant metastases (33.1% vs. 4.99%, P<0.001) compared to the cohort from the SUFAH (**Table 1**). This is primarily attributed to the inclusion of more ICU patient data in the MIMIC-IV database.

Furthermore, significant differences were observed between the two patient groups in laboratory parameters including CRP, PLA, NEUT, WBC, TP, ALB, TC, FIB, CEA, NLR, PLR, PIV, PNI, and SII (P < 0.001). Only LYM (P = 0.154), MON (P = 0.637), and HGB (P = 0.018) showed no statistically significant differences. In terms of treatment, the proportion of patients undergoing surgical intervention at the SUFAH (96.3% vs. 58.1%, P<0.001) was significantly higher than in the MIMIC cohort, while the proportions of radiotherapy and chemotherapy showed no significant difference (P>0.1). Prognostic scores, including the NPS and GPS, also exhibited significant differences between the two groups (P < 0.001). These results indicate that the external validation dataset and the internal dataset of the study showed significant statistical differences in the distribution of patient characteristics, providing stringent external validation conditions for assessing the model’s generalization capability. After splitting the MIMIC dataset into a 7:3 training and testing set, no significant differences were observed in baseline features between the two groups, validating the rationality and distribution consistency of the data split (**Table 2**).

**Table 2:** Comparison of baseline features between training and test sets in the MIMIC dataset partitioning.

Characteristic	Training set (n=1433)	Test set (n=616)	*P*
Survival status			0.908
Survival	857 (59.8%)	366 (59.4%)	
Death	576 (40.2%)	250 (40.6%)	
Overall Survival (days)	1976 (1736)	1988 (1741)	0.892
Age (years)	66.6 (15.0)	66.9 (14.7)	0.617
Gender			0.948
Male	753 (52.5%)	322 (52.3%)	
Female	680 (47.5%)	294 (47.7%)	
Race			0.93
White	1015 (70.8%)	447 (72.6%)	
Black	178 (12.4%)	74 (12.0%)	
Yellow	101 (7.05%)	41 (6.66%)	
Other	71 (4.95%)	26 (4.22%)	
Unknown	68 (4.75%)	28 (4.55%)	
ACCI			0.62
0 (<5)	319 (22.3%)	148 (24.0%)	
1 (5-7)	542 (37.8%)	222 (36.0%)	
2 (>=8)	572 (39.9%)	246 (39.9%)	
BMI (kg/m^2^)	27.4 (7.02)	28.4 (14.0)	0.13
T stage			0.17
T1	360 (25.1%)	183 (29.7%)	
T2	327 (22.8%)	125 (20.3%)	
T3	415 (29.0%)	171 (27.8%)	
T4	331 (23.1%)	137 (22.2%)	
N stage			0.738
N0	780 (54.4%)	351 (57.0%)	
N1	329 (23.0%)	137 (22.2%)	
N2	324 (22.6%)	128 (20.8%)	
M stage			0.788
M0	955 (66.6%)	415 (67.4%)	
M1	478 (33.4%)	201 (32.6%)	
CRP (mg/L)	50.2 (59.3)	49.9 (59.9)	0.928
LYM (×10^9^/L)	1.33 (2.98)	1.28 (0.80)	0.555
PLA (×10^9^/L)	263 (115)	256 (113)	0.159
NEUT (×10^9^/L)	7.34 (4.24)	6.84 (3.50)	0.009
MON (×10^9^/L)	0.68 (0.50)	0.67 (0.45)	0.722
WBC (×10^9^/L)	9.65 (5.34)	9.11 (3.78)	0.01
TP (g/dL)	5.85 (0.96)	5.81 (0.97)	0.488
ALB (g/dL)	3.15 (0.63)	3.17 (0.66)	0.545
HGB (g/dL)	11.6 (1.81)	11.7 (1.77)	0.235
TC (mg/dL)	139 (49.3)	141 (48.9)	0.596
FIB (mg/dL)	407 (174)	402 (170)	0.566
CEA (ng/mL)	46.0 (27.76)	45.9 (26.09)	0.996
NLR	9.26 (10.4)	8.78 (11.8)	0.428
PLR	328 (322)	315 (391)	0.486
LMR	2.85 (4.83)	2.77 (3.19)	0.698
PIV	1730 (2556)	1671 (3871)	0.764
PNI	31.6 (6.36)	31.7 (6.77)	0.794
SII	2543 (3441)	2506 (5401)	0.888
Surgery			0.957
No	599 (41.8%)	259 (42.0%)	
Yes	834 (58.2%)	357 (58.0%)	
Radiotherapy			0.712
No	1257 (87.7%)	536 (87.0%)	
Yes	176 (12.3%)	80 (13.0%)	
Chemotherapy			0.324
No	1213 (84.6%)	510 (82.8%)	
Yes	220 (15.4%)	106 (17.2%)	
NPS			0.322
Low (0)	4 (0.49%)	4 (1.14%)	
Medium (1)	106 (12.9%)	50 (14.2%)	
High (2)	714 (86.7%)	297 (84.6%)	
GPS			0.222
Low (0)	172 (15.0%)	73 (15.3%)	
Medium (1)	426 (37.2%)	197 (41.4%)	
High (2)	547 (47.8%)	206 (43.3%)	

### 3.2 Feature selection

**Figure 3:**
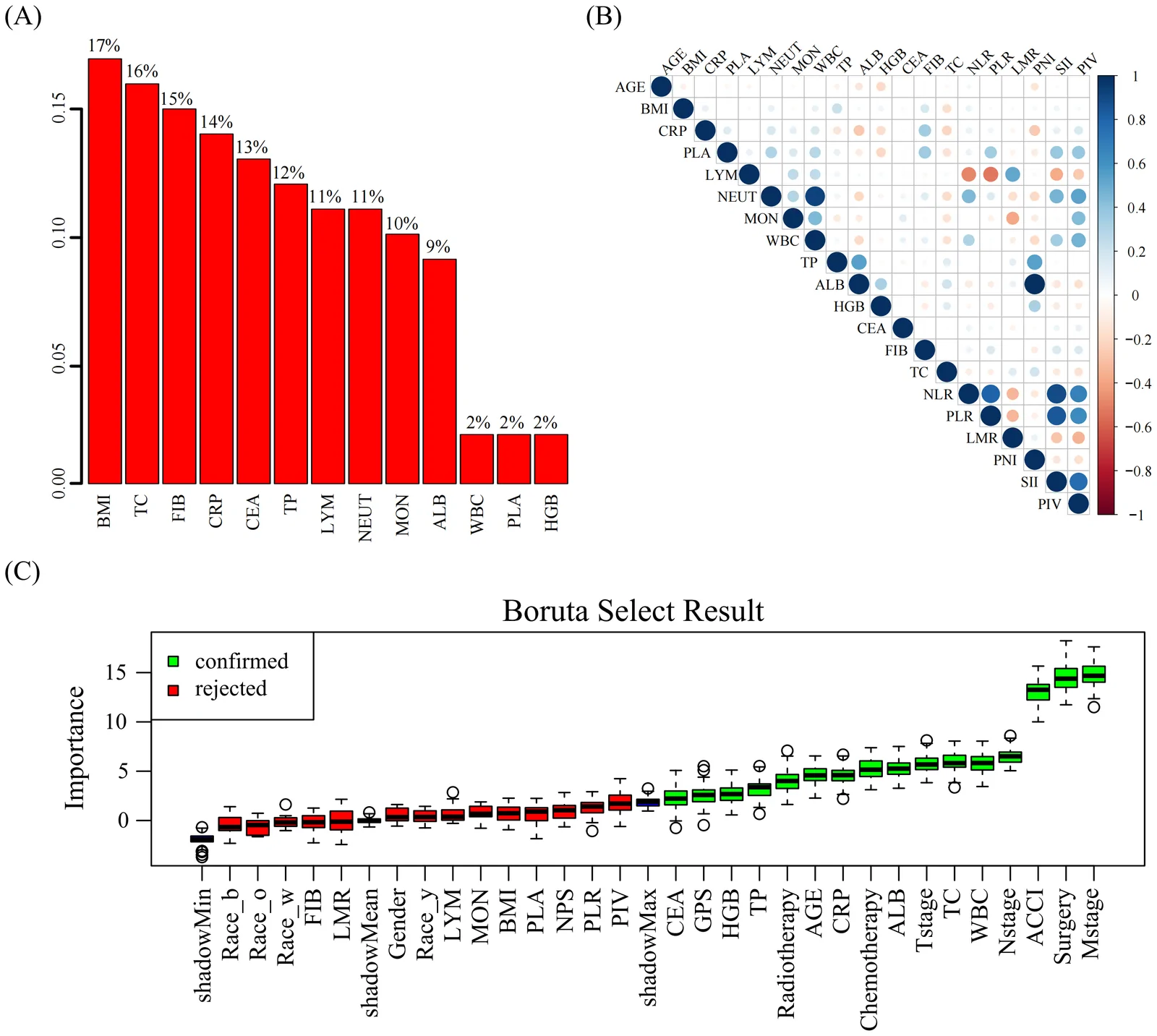
Feature screening results. **(A)** Bar chart of missing value proportions for each feature; **(B)** Heatmap of feature correlations; **(C)** Ranking and classification of feature importance using the Boruta algorithm (confirmed/rejected).

To ensure the effectiveness of model training, the study conducted a detailed feature screening. In the MIMIC dataset, the proportion of missing values for 9 features, including BMI and TC, exceeded 10%, but the proportion of missing values for all features did not exceed 20% (**Figure 3A**) (5). In the dataset from the SUFAH, the proportion of missing values was consistently below 5% (5). Feature correlation analysis revealed that the Pearson correlation coefficients for 6 feature groups, such as ALB and PNI, were greater than 0.6, indicating strong correlations (**Figure 3B**). To avoid the impact of multicollinearity on model performance, the study removed four strongly correlated and collinear features: NEUT, NLR, PNI, and SII.

The feature importance ranking of the Boruta algorithm revealed that Mstage, Surgery, and ACCI ranked as the top three features. Ultimately, 16 “confirmed” features were selected, including tumor stage (Tstage, Nstage, Mstage), treatment modality (Surgery, Chemotherapy, Radiotherapy), and inflammatory and nutritional biomarkers (CRP, ALB, GPS) (**Figure 3C**). These features were considered core drivers of the model’s predictive power, comprehensively reflecting the impact of disease progression, treatment efficacy, and overall patient status on prognosis (21).

### 3.3 Model training and evaluation

**Figure 4:**
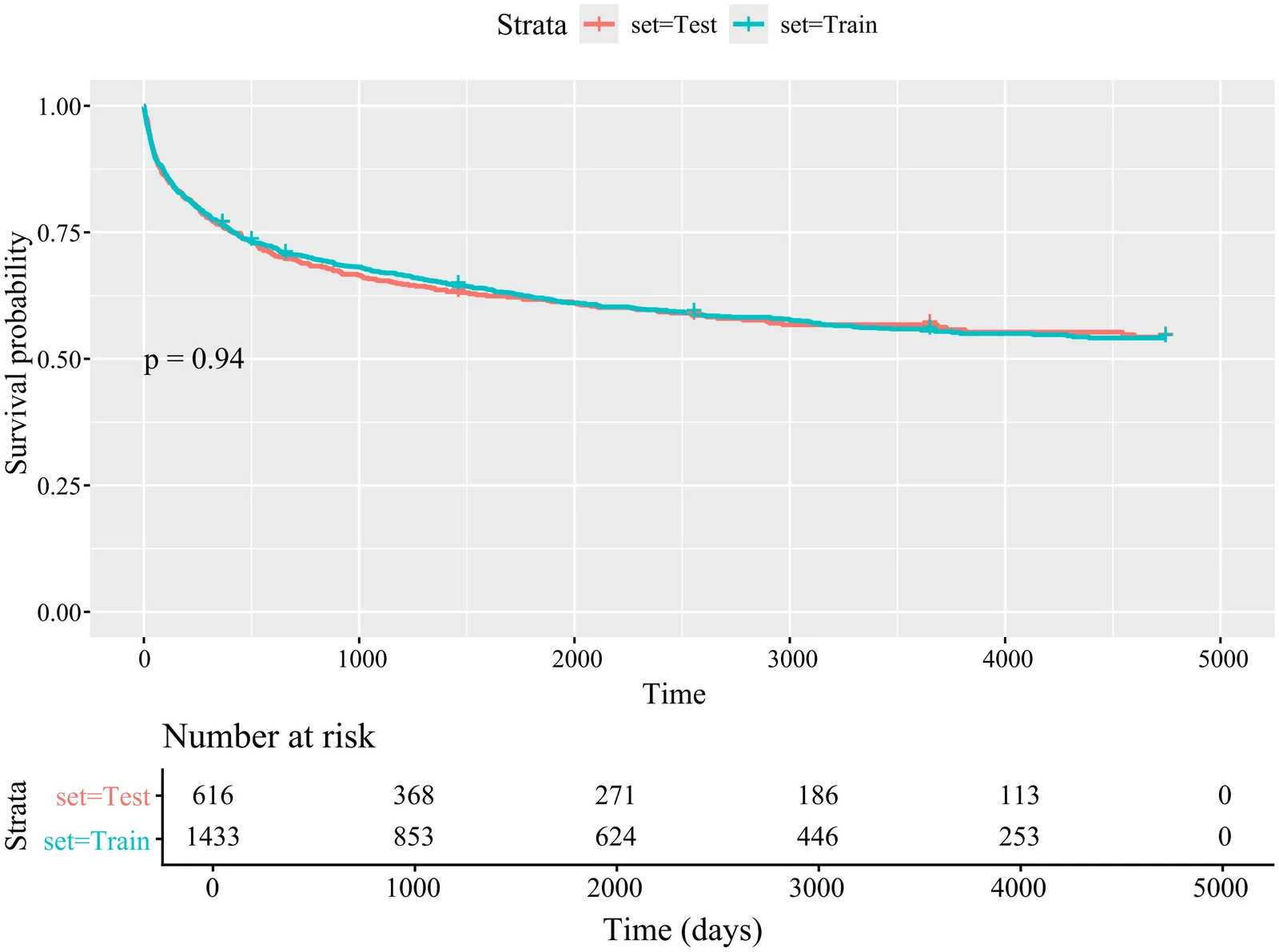
Kaplan-Meier survival curves for the training and test sets of the MIMIC dataset, with log-rank test results to verify group balance.

The rigorous model training and evaluation process first validated the rationality of the training and test set data partition. Kaplan-Meier survival curves demonstrated that the training and test sets derived from the MIMIC dataset exhibited nearly identical trends in survival probability over time. The Log-rank test yielded a P-value of 0.94, indicating no statistically significant difference in survival outcomes between the two groups (**Figure 4**). This provides a solid foundation for subsequent survival analysis and model validation.

Through hyperparameter search, the optimal parameter settings for the nine algorithms are as follows: DT model alpha=0.1, minbucket=5; RSF model ntree=500, mtry=5, nodesize=15; Cox model use_weights=‘use’; Lasso regression alpha=1, using the “lambda.1se” criterion to select regularization parameters, with the consistency index (C-index) as the metric; Ridge regression alpha=0, using the “lambda.min” criterion; EN sets alpha=0.45, using the “lambda.1se” criterion; XGB sets nrounds=384, max_depth=1, eta=0.1378; LGBM sets n.trees=100, interaction_depth=3, n.minobsinnode=5, shrinkage=0.0505. The Stack integration algorithm uses RSF, XGB, and Cox as base learners, with logistic regression as the meta learner for fusion. The final model is retrained using the optimized hyperparameters and evaluated on both internal and external validation sets (5).

#### 3.3.1 Stepwise incremental-value analysis results

The stepwise expansion from TNM-only to the full multidimensional model demonstrated progressive improvement in prognostic discrimination across all evaluation metrics (**Table 3**; **Supplementary Figure 2**). On the external validation set, the TNM-only model (Model 0) achieved a mean AUROC of 0.664 (95% CI: 0.569–0.748) and surv.cindex of 0.631 (95% CI: 0.583–0.686), consistent with known limitations of anatomical staging alone (1). Addition of demographics and comorbidity (Model 1 vs. Model 0): Incorporating Age and ACCI yielded substantial improvement, with mean AUROC increasing to 0.741 (Δ +0.077, DeLong test Z = 6.42, P < 0.001) and surv.cindex rising to 0.712 (Δ +0.081). This increment underscores the prognostic relevance of baseline physiological reserve independent of tumor anatomy (2). Addition of treatment variables (Model 2 vs. Model 0): The TNM + Treatment model achieved mean AUROC 0.783 (Δ +0.119 vs. Model 0, Z = 8.91, P < 0.001), with surv.cindex 0.758 (Δ +0.127). Notably, direct comparison between Model 2 and Model 1 (AUROC 0.783 vs. 0.741, Z = 3.87, P < 0.001) indicated that treatment modality information provided additional prognostic value beyond demographics and comorbidity. However, we emphasize that treatment variables in this retrospective design reflect treatment assignment patterns rather than causal therapeutic effects; the observed associations may be confounded by indication and patient selection (3). Addition of laboratory biomarkers (Model 3 vs. Model 0): The TNM + Laboratory model attained mean AUROC 0.696 (Δ +0.032 vs. Model 0, Z = 2.14, P = 0.032), with modest incremental value compared with demographic or treatment domains. This model underperformed Model 1 and Model 2, suggesting that laboratory markers alone, without contextual comorbidity or treatment information, provide limited prognostic enhancement over anatomical staging in this cohort (4). Full model integration (Model 4 vs. all predecessors): The complete 16-feature RSF model achieved mean AUROC 0.818 (95% CI: 0.762–0.866), representing increments of +0.154, +0.077, +0.035, and +0.122 over Models 0–3, respectively (all DeLong test P < 0.001) (**Supplementary Table 3**). The surv.cindex reached 0.779 (95% CI: 0.737–0.811), with respective increments of +0.148, +0.067, +0.021, and +0.062 (**Table 3**). The Brier score improved to 0.120, indicating superior calibration. The sequential ΔAUROC and Δsurv.cindex patterns (**Supplementary Figure 2**) demonstrate that each feature domain contributes non-redundant prognostic information, with demographics/comorbidity and treatment modalities accounting for the largest individual increments, and the full integration achieving synergistic enhancement. Sensitivity analysis excluding treatment variables (RSF No Treat, 13 features) yielded mean AUROC 0.814 (Δ −0.004 vs. Full RSF), confirming that the model’s discriminative capacity is not dependent on treatment information alone.

**Table 3:** Stepwise incremental-value analysis of the RSF model hierarchy on external validation.

Model	Feature domains	N features	External validation set				Incremental vs. immediate predecessor		
			mean AUROC (95% CI)	mean AUPR	mean Brier	surv.cindex (95% CI)	ΔAUROC	Z (DeLong)	P value
Model 0	TNM only	3	0.664 (0.569–0.748)	0.312	0.198	0.631 (0.583–0.686)	—	—	—
Model 1	TNM + Age/ACCI	5	0.741 (0.672–0.803)	0.398	0.165	0.712 (0.668–0.754)	0.077	6.42	<0.001
Model 2	TNM + Treatment	6	0.783 (0.726–0.834)	0.456	0.145	0.758 (0.718–0.796)	0.042	3.87	<0.001
Model 3	TNM + Laboratory	10	0.696 (0.646–0.743)	0.356	0.182	0.717 (0.661–0.758)	−0.087	−5.21	<0.001
Model 4	Full RSF	16	0.818 (0.762–0.866)	0.543	0.12	0.779 (0.737–0.811)	0.122	7.68	<0.001

ΔAUROC represents change versus immediate predecessor model. Model 3 negative increment indicates laboratory-only addition underperforms relative to Model 2 (TNM+Treatment). All DeLong tests two-sided. AUPR = area under precision-recall curve.

#### 3.3.2 Sensitivity to case inclusion criteria

To assess whether the inclusion of cases without accessible pathology reports (n=802, 39.1%) influenced model performance, we conducted a sensitivity analysis restricting the training set to ICD-pathology concordant cases only (n=1,175). The Model 4 (Full RSF) retrained on this restricted cohort achieved external validation AUROC 0.817 (95% CI: 0.761–0.865), virtually identical to the primary analysis (AUROC 0.818, Δ−0.001). Stepwise incremental patterns were preserved: Model 1 ΔAUROC +0.076, Model 2 ΔAUROC +0.043, Model 3 ΔAUROC −0.086, Model 4 ΔAUROC +0.121 (**Supplementary Table 6**). These findings confirm that the hierarchical incremental-value framework is robust to case inclusion criteria.

#### 3.3.3 Performance on the training set

**Figure 5:**
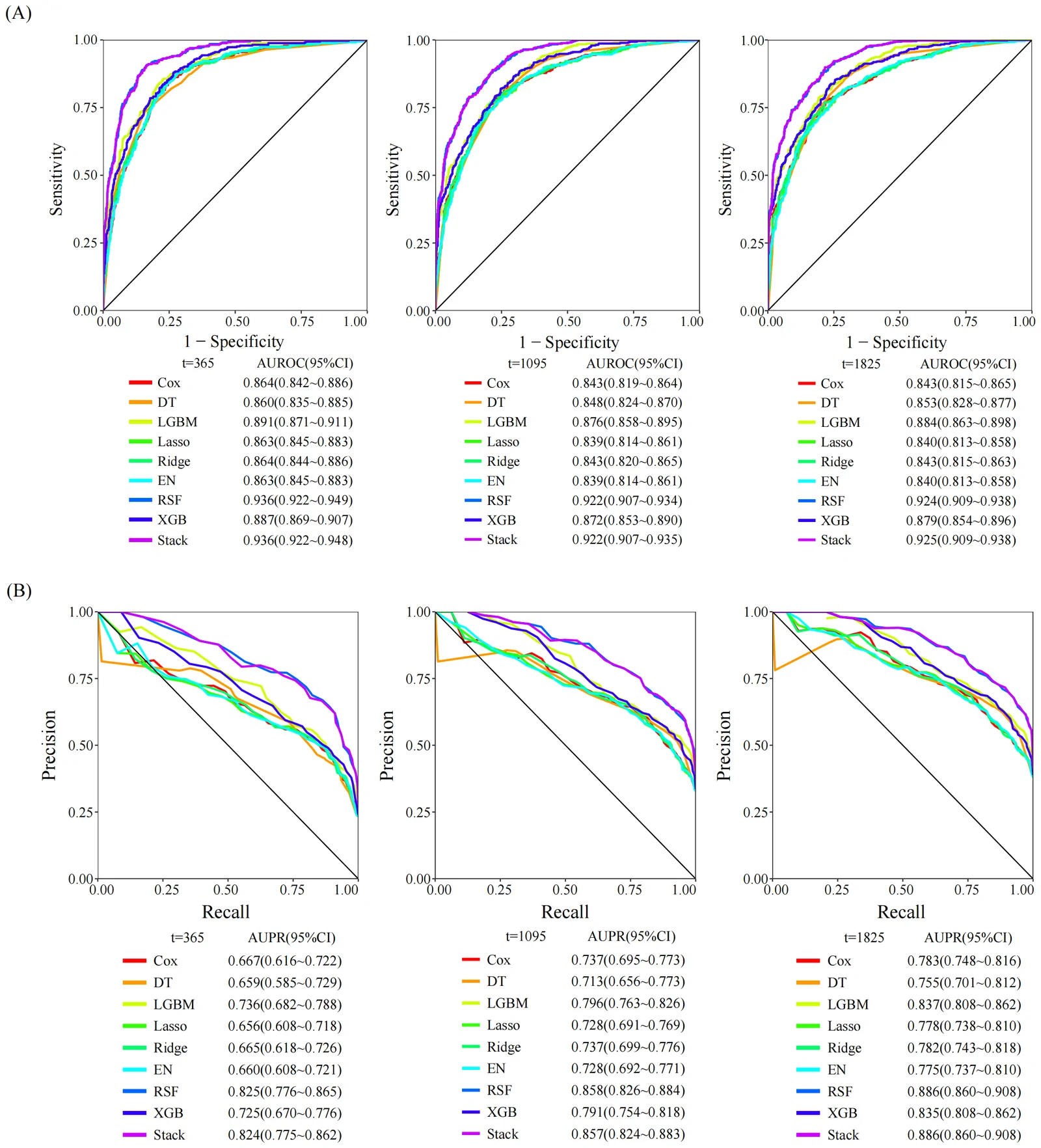
Performance of each model on the training set (1). **(A)** ROC curves at t=365, 1095, and 1825 days; **(B)** PR curves at corresponding time points.

**Figure 6:**
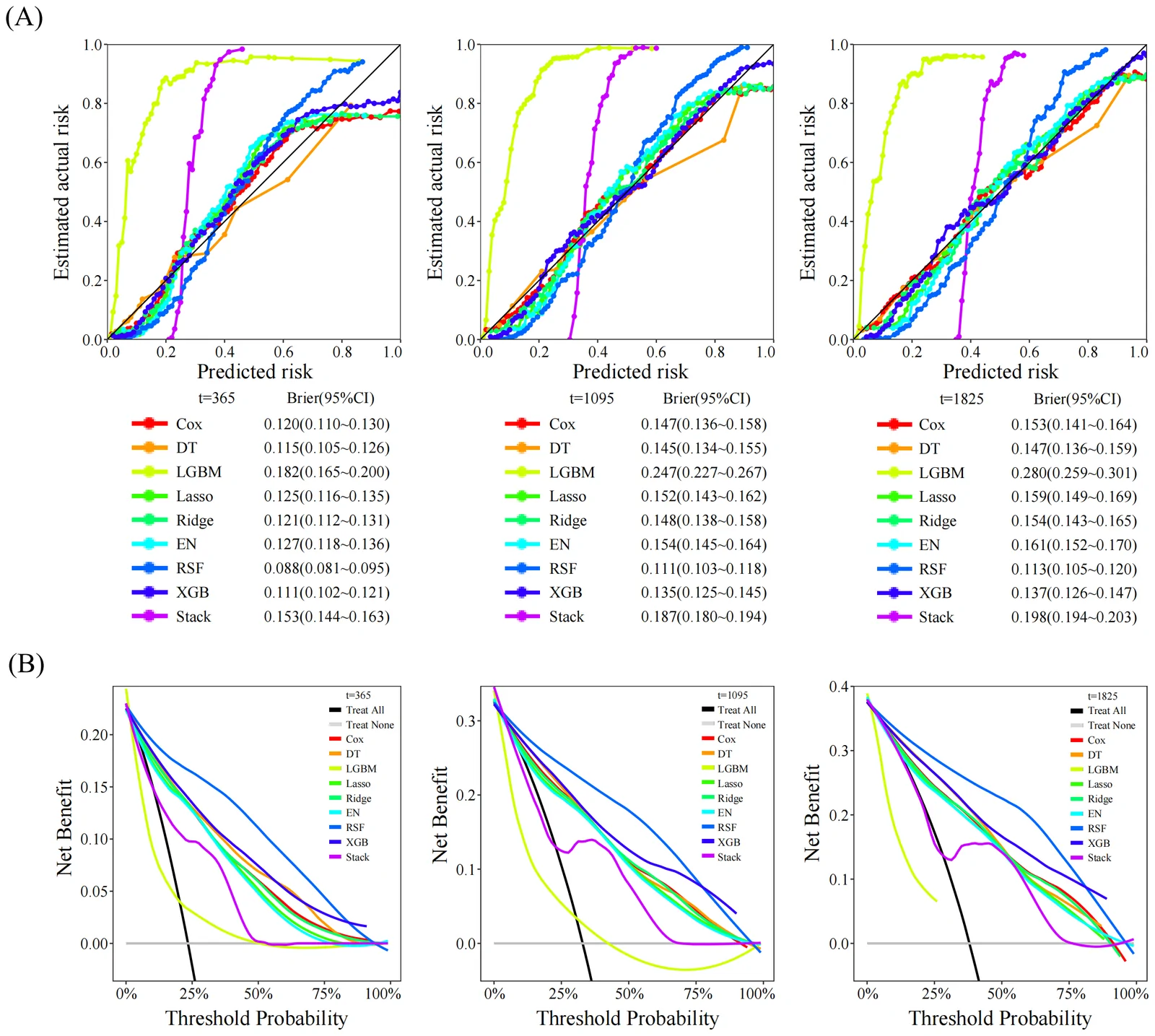
Performance of each model on the training set (2). **(A)** Calibration curves at t=365, 1095, and 1825 days; **(B)** Decision curves at corresponding time points.

The RSF model showed the highest comprehensive score on the training set; however, training set metrics may overestimate generalization performance. ROC curve analysis revealed that all models exhibited strong discriminative ability, with the RSF model and Stack model performing optimally. The AUROC values of the RSF model at t=365 days, t=1095 days, and t=1825 days were 0.936 (95% CI: 0.922-0.949), 0.922 (95% CI: 0.907-0.934), and 0.924 (95% CI: 0.909-0.938), respectively (**Figure 5A**). PR curve results indicated that the AUPR values of the RSF model at t=365 days, t=1095 days, and t=1825 days were 0.825 (95% CI: 0.776-0.865), 0.858 (95% CI: 0.826-0.884), and 0.886 (95% CI: 0.860-0.908), respectively (**Figure 5B**). Calibration curve analysis showed that the RSF model exhibited the lowest Brier score (0.088 at t = 365 days, 0.111 at t = 1095 days, and 0.113 at t = 1825 days) among compared algorithms, though calibration assessment requires external validation between predicted and actual risks (**Figure 6A**). DCA curves demonstrated that the RSF model achieved a relatively higher net benefit in the training set, though DCA results from training data may overestimate clinical utility due to overfitting within the clinically common threshold probability range of 20% to 80% in the training set. However, DCA results from the training set should be interpreted cautiously, as they may overestimate clinical utility due to overfitting. External validation DCA curves showed modest net-benefit differences among models, suggesting that the incremental clinical utility of RSF over alternative models requires further evaluation in prospective studies (**Figure 6B**) (25, 26).

#### 3.3.4 Test set performance

**Figure 7:**
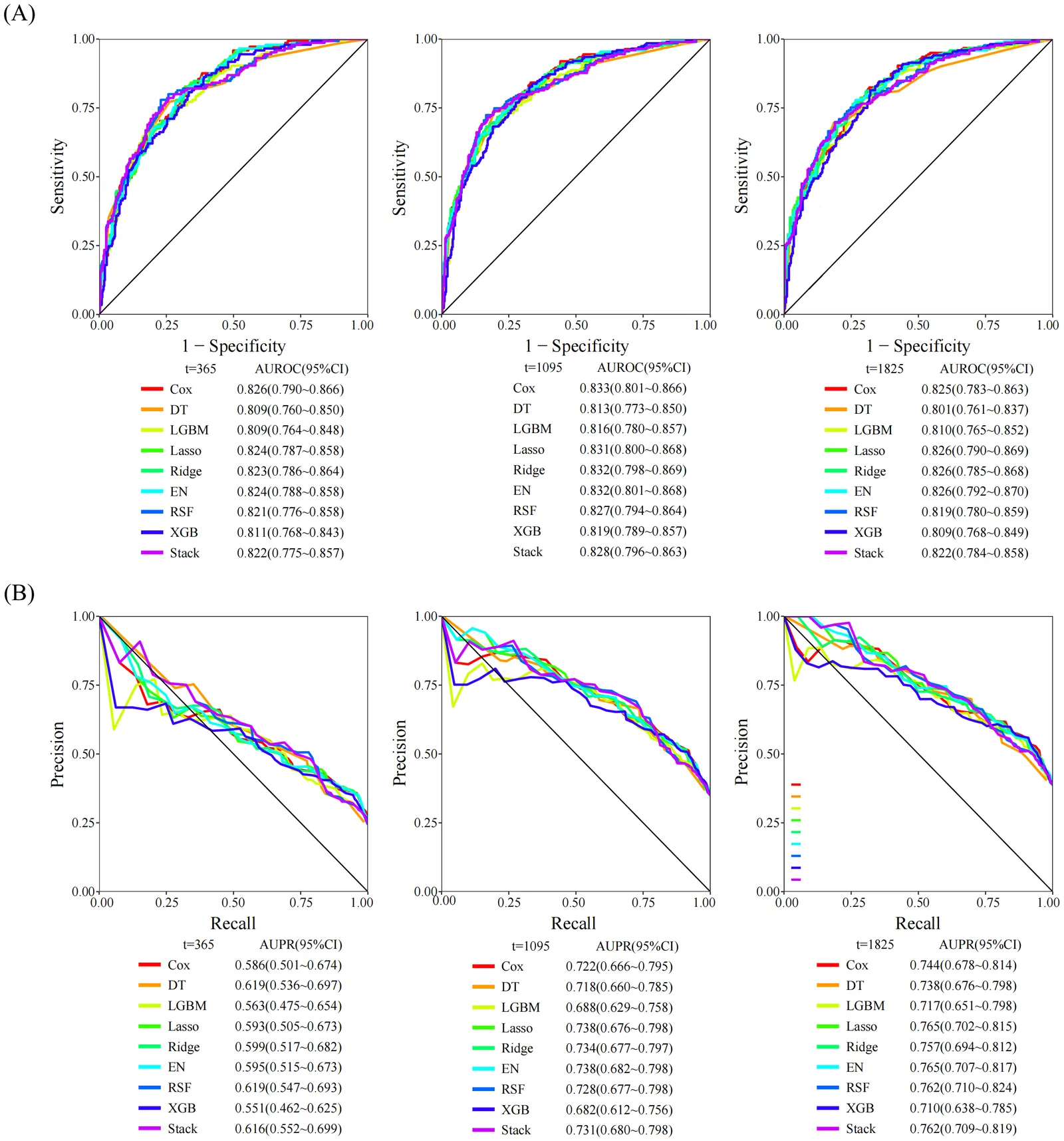
Performance of each model on the test set (internal validation) (1). **(A)** ROC curves at t=365, 1095, and 1825 days; **(B)** PR curves at corresponding time points.

**Figure 8:**
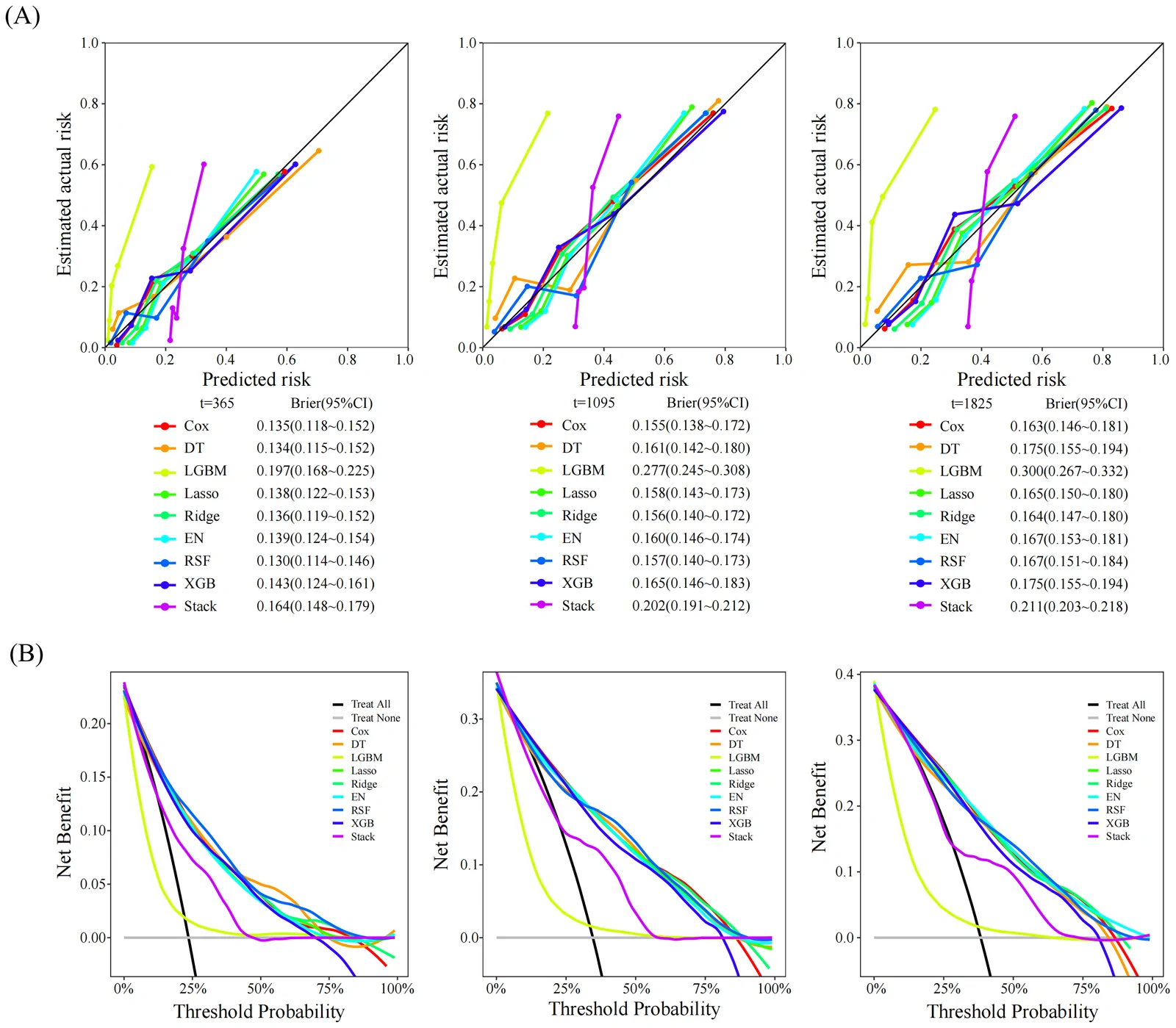
Performance of each model on the test set (internal validation) (2). **(A)** Calibration curves at t=365, 1095, and 1825 days; **(B)** Decision curves at corresponding time points.

The results of the test set (internal validation) demonstrated a trend distinct from that of the training set. Traditional linear models showed higher AUROC than tree-based models in this internal validation setting. The Cox model achieved the best AUROC performance, with AUROC values of 0.826 (95% CI: 0.790-0.866), 0.833 (95% CI: 0.801-0.866), and 0.825 (95% CI: 0.783-0.863) at time points t=365 days, t=1095 days, and t=1825 days, respectively, with an average AUROC of 0.828 across all time points. The EN model ranked second, with an average AUROC of 0.827 (**Figure 7A**). In terms of AUPR, the Stack model performed best, with an average AUPR of 0.699 across all time points, while the RSF model was on par (**Figure 7B**). For the Brier score, the RSF model showed the best performance, with an average Brier score of 0.150 across all time points, matching the Cox model (**Figure 8A**). The comprehensive score indicated that the EN model ranked first with a score of 0.954, followed by the Lasso, Ridge, RSF, and Cox models in the top five, consistent with potential advantages of linear models in controlling overfitting in smaller sample settings, though this pattern reversed in external validation (**Table 4**).

**Table 4:** Comprehensive score of each model on test set indicators.

Model	mean_AUROC	mean_AUPR	mean_Brier	norm_AUROC	norm_AUPR	norm_Brier	Score
EN	0.827	0.699	0.155	0.967	0.934	0.961	0.954
Lasso	0.827	0.699	0.154	0.939	0.919	0.976	0.945
Ridge	0.827	0.697	0.152	0.951	0.887	0.994	0.944
RSF	0.822	0.703	0.151	0.726	0.998	0.998	0.907
Cox	0.828	0.684	0.151	1.000	0.657	1.000	0.886
Stack	0.824	0.703	0.192	0.794	1.000	0.617	0.804
DT	0.808	0.692	0.156	0.000	0.793	0.951	0.581
XGB	0.813	0.648	0.161	0.274	0.000	0.909	0.394
GBM	0.812	0.656	0.258	0.204	0.151	0.000	0.119

The DCA curve further analyzes the clinical application value of each model at different time points (**Figure 8B**). Short-term prediction (t=365 days): Within the threshold range of 10%~30%, the RSF or XGB model showed higher net benefit within this threshold range with stronger curve stability. Medium-term prediction (t=1095 days): Both RSF and XGB exhibit optimal performance within the threshold range of 10%~40%, featuring high net benefit and low volatility, making them suitable for medium-term (3-year) risk stratification. Long-term prediction (t=1825 days): The RSF model achieves the highest and most stable net benefit within the threshold range of 10%~50%, suggesting potential applicability for long-term risk stratification, subject to external validation (27).

#### 3.3.5 Performance of the external validation set

**Figure 9:**
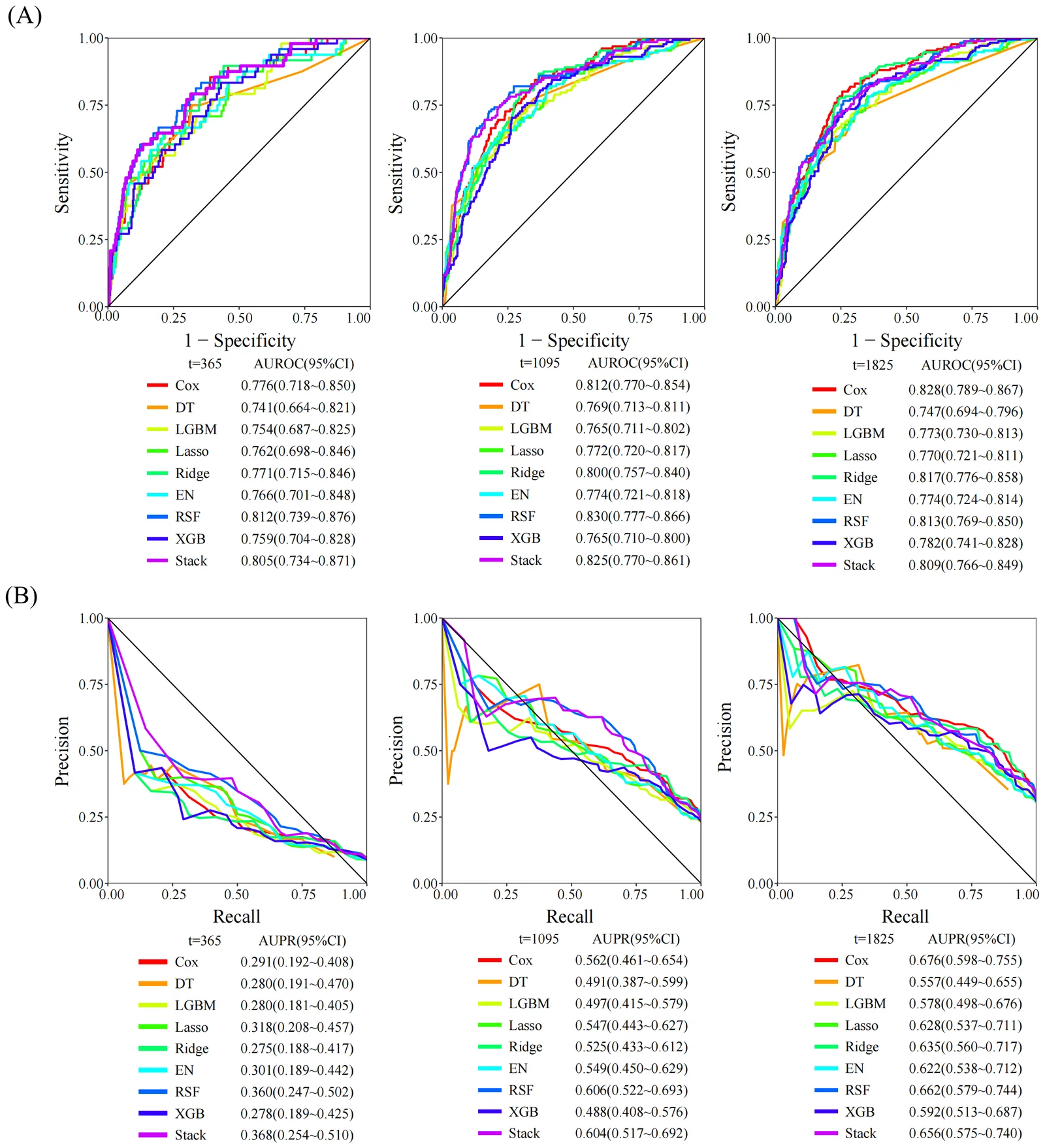
Performance of each model on the external validation set (1). **(A)** ROC curves at t = 365, 1095, and 1825 days; **(B)** PR curves at corresponding time points.

**Figure 10:**
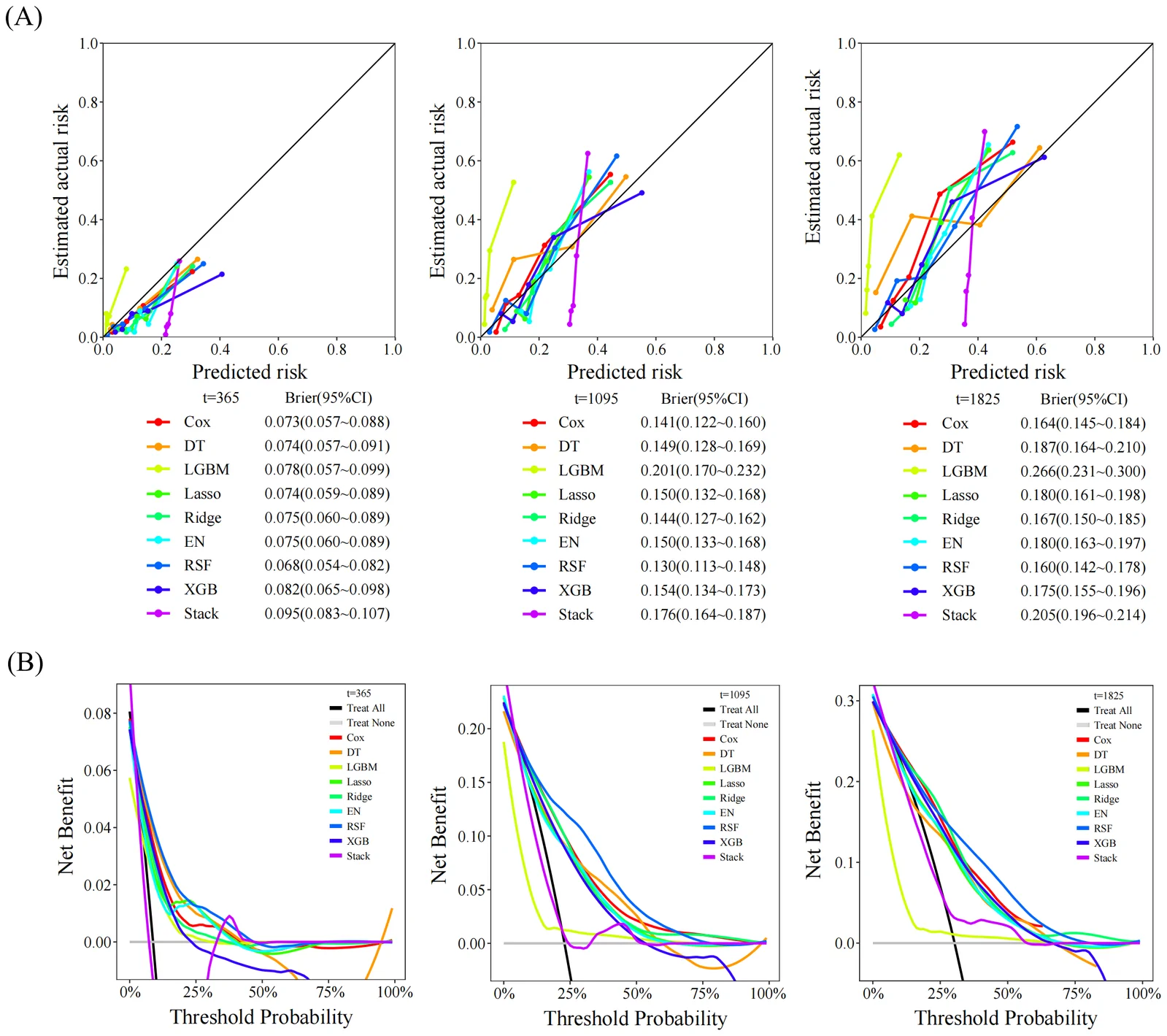
Performance of each model on the external validation set (2). **(A)** Calibration curves at t=365, 1095, and 1825 days; **(B)** Decision curves at corresponding time points.

The results of the external validation set (independent cohort) differed from those of the test set, demonstrating that tree-based models and ensemble models showed more stable performance rankings across internal and external validation compared with traditional linear models (27). The RSF model performed best in terms of AUROC (**Figure 9A**), with AUROC values of 0.812 (95% CI: 0.739-0.876), 0.830 (95% CI: 0.777-0.866), and 0.813 (95% CI: 0.769-0.850) at time points t=365 days, t=1095 days, and t=1825 days, respectively, with an average AUROC of 0.818 across all time points. The Cox model ranked second, with an average AUROC of 0.805. In terms of AUPR, the RSF model performed best, with an average AUPR of 0.543 across all time points, while the Stack model was tied (**Figure 9B**). Regarding the Brier score, the RSF model also showed the best performance, with an average Brier score of 0.120 across all time points, followed by the Cox model (**Figure 10A**).

Notably, the EN model that performed best on the test set achieved only 0.771 AUROC on the external validation set, ranking sixth, suggesting reduced performance stability when applied to the independent cohort. The comprehensive score shows that the RSF model topped the list with the highest score of 1.000, followed by the top five models in order: RSF, Cox, Stack, Ridge, and Lasso (**Table 5**). This is consistent with observations that tree-based ensemble methods can reduce overfitting variance in this specific external validation setting (27).

**Table 5:** Comprehensive score of each model on the external validation set.

Model	mean_AUROC	mean_AUPR	mean_Brier	norm_AUROC	norm_AUPR	norm_Brier	Score
RSF	0.818	0.543	0.120	1.000	1.000	1.000	1.000
Cox	0.805	0.510	0.126	0.803	0.673	0.897	0.791
Stack	0.813	0.543	0.159	0.921	0.999	0.368	0.763
Ridge	0.796	0.479	0.129	0.661	0.362	0.851	0.625
Lasso	0.768	0.498	0.134	0.238	0.551	0.762	0.517
EN	0.771	0.490	0.135	0.290	0.479	0.751	0.507
XGB	0.768	0.453	0.137	0.244	0.101	0.719	0.355
DT	0.752	0.442	0.136	0.000	0.000	0.727	0.242
GBM	0.764	0.452	0.181	0.178	0.093	0.000	0.091

It is important to note that this comprehensive score is a normalized composite ranking metric used for model comparison within this study, not an absolute measure of predictive accuracy. A score of 1.000 indicates the highest rank among the compared models, not perfect prediction performance.

DCA curve analysis revealed (**Figure 10B**): For short-term projections (t=365 days), the RSF and DT curves showed significant overlap at the 10%-30% threshold, with low net benefits ranging between 0.01-0.06. In medium-term projections (t=1095 days), all models showed higher absolute net benefits compared to the 365-day period. The RSF maintained a relatively stable net benefit of 0.10–0.20 within the 5%-35% threshold range, with reduced fluctuation across threshold values. For long-term projections (t=1825 days), the RSF peaked at 0.30 (threshold ≈5%), with the highest net benefits observed in the 30%-60% threshold range (0.05–0.15) (27).

Statistical comparison of model performance: Paired comparisons between RSF and other models were performed using the DeLong test for AUROC differences and the bootstrap method for Brier score differences. In the external validation set, RSF showed statistically significantly higher AUROC than XGB (P = 0.012), LGBM (P = 0.008), and DT (P < 0.001), but the differences from Cox (P = 0.142) and Stack (P = 0.198) did not reach statistical significance (**Supplementary Table 4**). These results suggest that while RSF demonstrated the most consistent overall performance, its advantages over some competing models were modest and should be interpreted with caution.

### 3.4 Model comprehensive evaluation and optimal model selection

**Figure 11:**
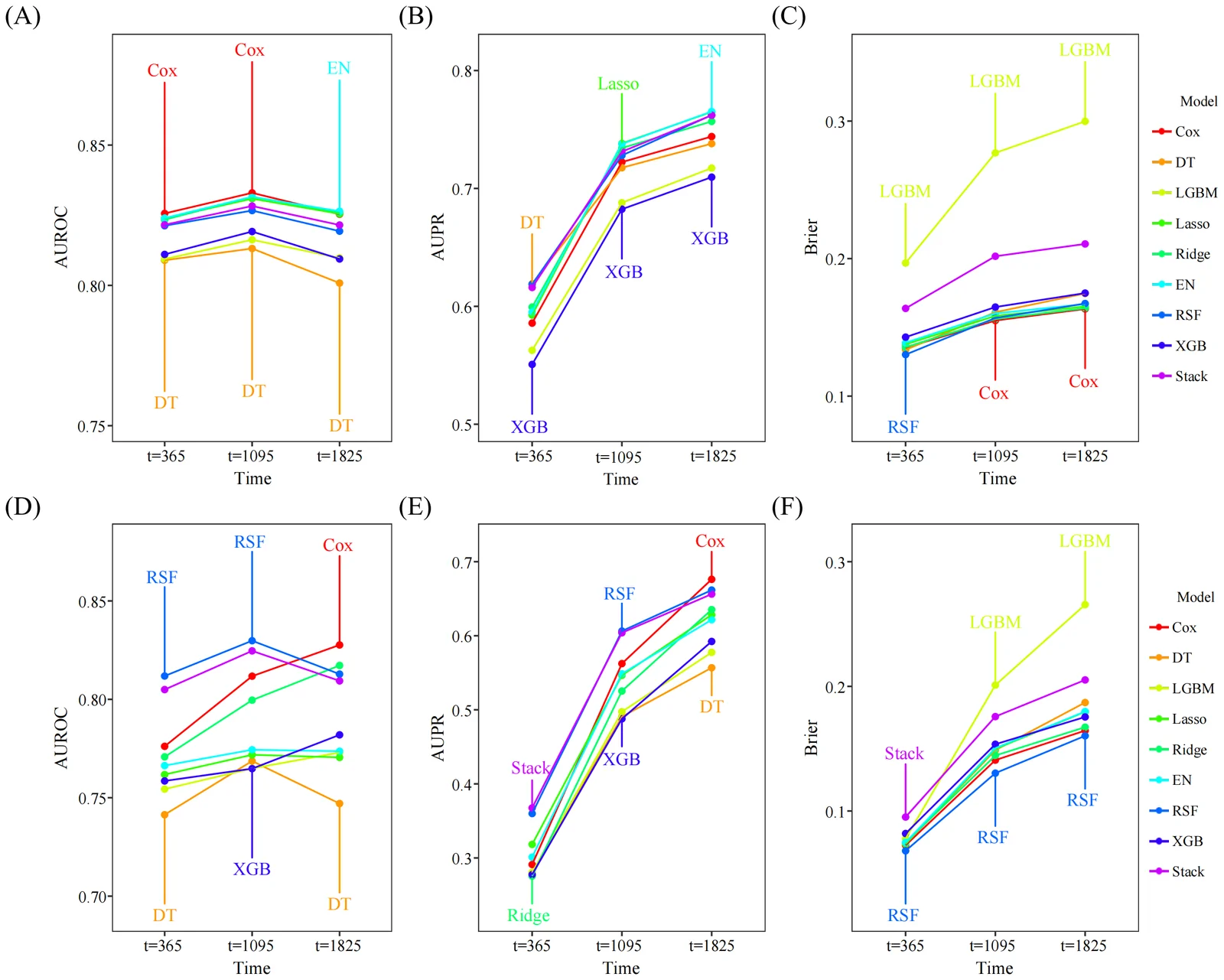
Comparison of AUROC, AUPR, and brier score for each model. **(A–C)** Comparison of AUROC, AUPR, and brier scores for each model on the test set; **(D–F)** Comparison of AUROC, AUPR, and brier scores for each model on the external validation set.

The performance of the comprehensive test set and external validation set demonstrated that the RSF model was selected as the final model based on prespecified selection criteria (**Figure 11**). In the external validation set, the RSF model achieved the highest AUROC, AUPR, and lowest Brier score among the nine compared algorithms (0.818, 0.543, and 0.120, respectively), and comprehensive score 1.000 (top rank among compared models) (**Table 5**). Although the AUROC of the RSF model in the test set (0.822) was slightly lower than that of EN, it remained within the top five and showed relatively smaller performance variation across time points at the 365 days, 1095 days, and 1825 days time points compared to other models (**Figure 7A**). In contrast, linear models (such as EN, Lasso, and Ridge) performed well in the test set but showed insufficient generalization ability in the external validation set. The custom Stack model, while demonstrating good comprehensive performance, was characterized by high complexity and weak interpretability. Therefore, the RSF model was selected as the final model due to its relatively balanced performance across training, internal validation, and external validation sets (25, 26).

The results of the surv.cindex further support the robustness of the RSF model (**Supplementary Table 5**). In the test set, the surv.cindex of all models decreased, but the RSF model (0.803, 95% CI: 0.778-0.828) remained optimal, with a relatively narrow confidence interval (0.050), indicating minimal performance fluctuation in internal validation. In the external validation set, the surv.cindex of the RSF model (0.779, 95% CI: 0.737-0.811) was significantly higher than that of other models, and its confidence interval (0.074) was the narrowest among all models, suggesting relatively stable discriminative performance in this independent cohort. In contrast, the confidence interval of linear models (e.g., EN, 0.737, 95% CI: 0.696-0.779) was wider (0.083), suggesting greater performance variation when applied to the independent cohort (27).

### 3.5 Model interpretation

As the final selected model, the RSF model’s feature importance analysis revealed the time-dependent effects of different factors on patient survival prognosis and their individual contribution patterns (24, 25).

#### 3.5.1 Time-dependent feature importance

**Figure 12:**
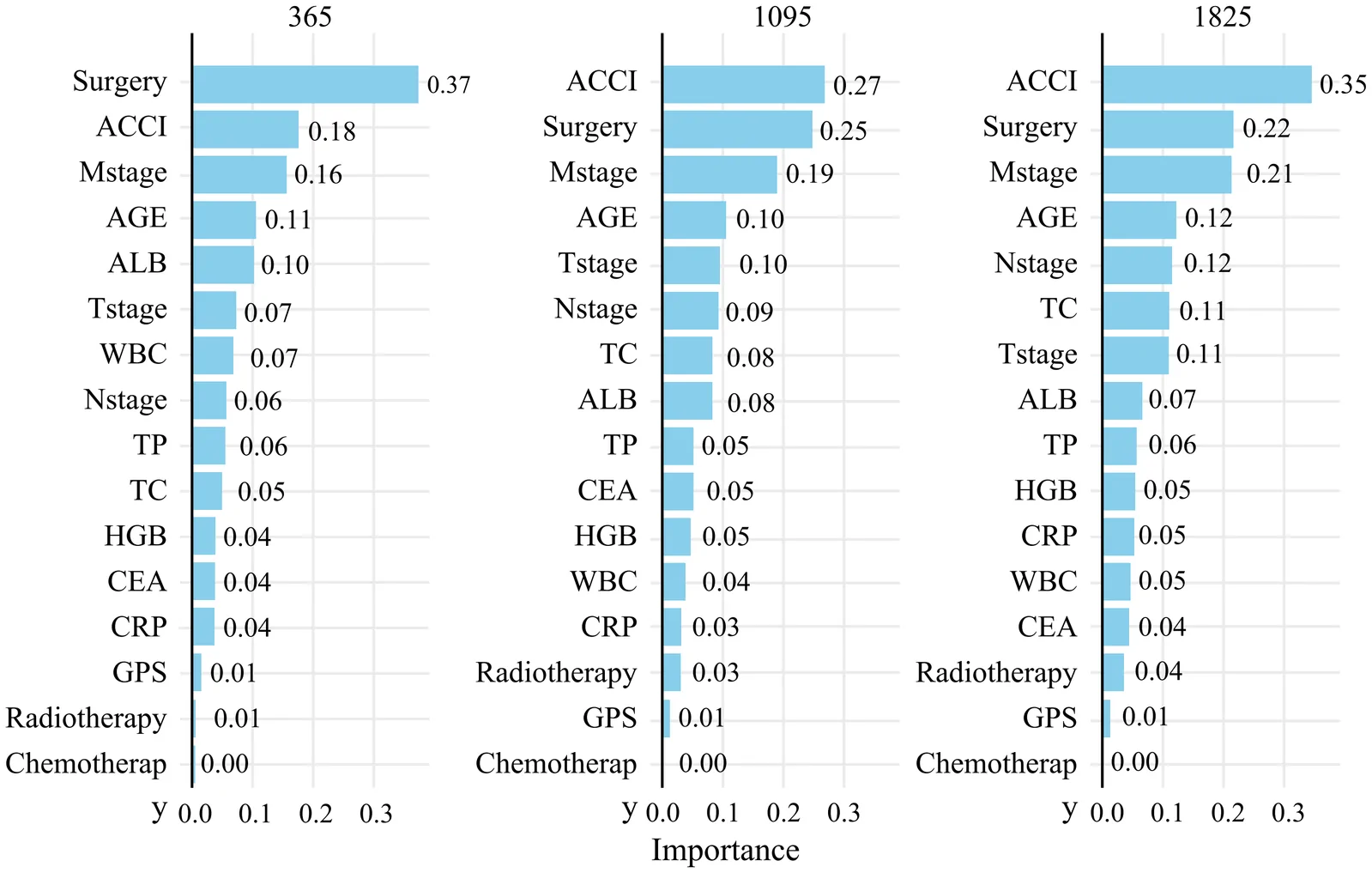
Bar chart illustrating feature importance at specific time points for the RSF model (based on permutation significance), showing the ranking of each feature’s contribution to model predictions at three follow-up time points: t = 365 days, t = 1095 days, and t = 1825 days (based on Brier score loss), with numerical values representing importance scores (Importance).

Significant differences were observed in the dominant prognostic features at different follow-up time points (365 days, 1095 days, 1825 days) (**Figure 12**). Short-term (365 days): Surgery was the most critical feature, followed by the ACCI and Mstage. This indicates that treatment interventions (e.g., Surgery) were associated with the largest model-predicted effects on short-term survival probability. Intermediate-term (1095 days) and Long-term (1825 days): ACCI replaced Surgery as the primary feature, with Surgery and Mstage ranking second and third, respectively. This indicates that the model assigned greater importance to baseline health status for long-term predictions and the extent of tumor metastasis (24, 25).

**Figure 13:**
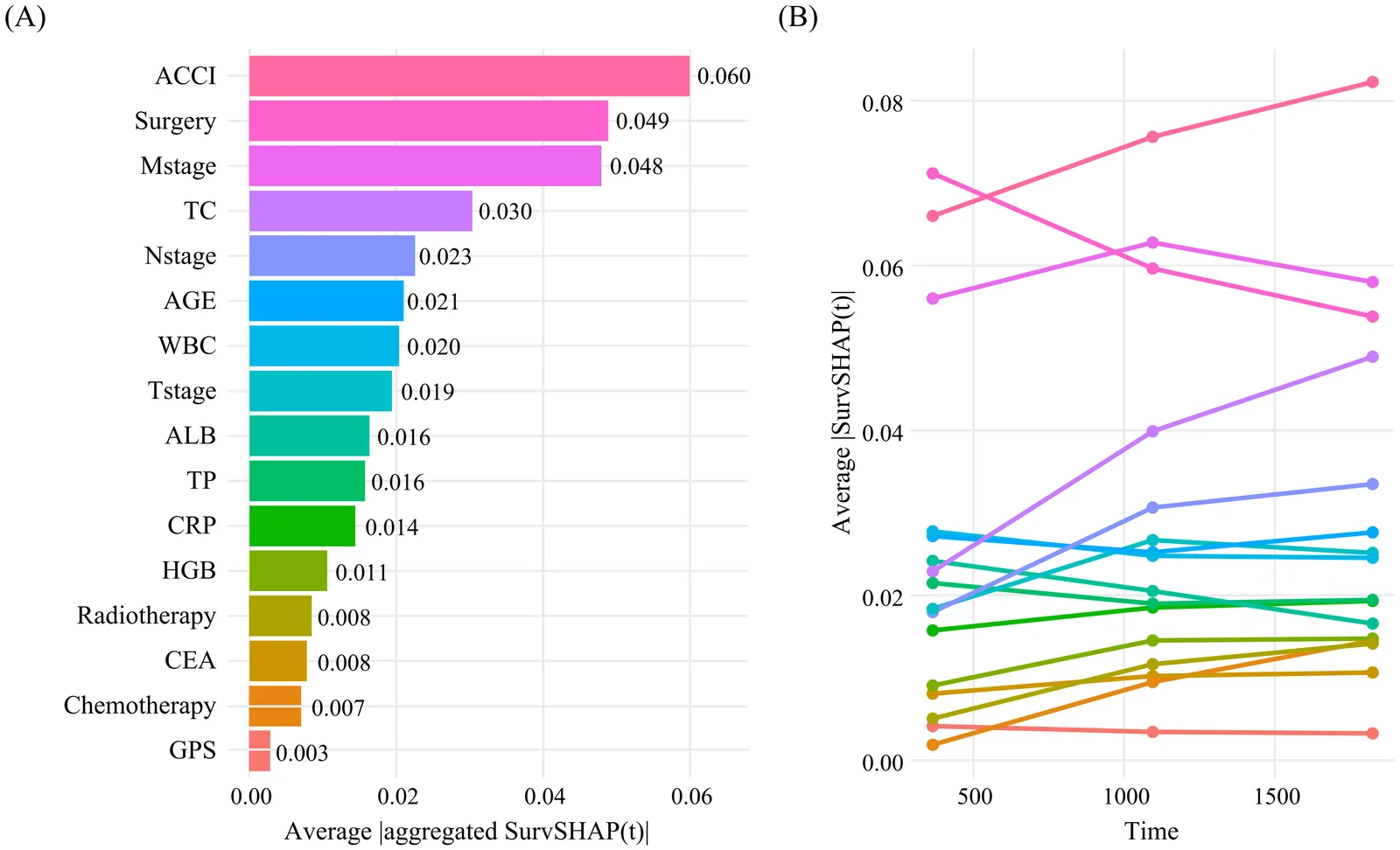
Time-dependent feature importance and SHAP analysis of the RSF model. **(A)** Overall feature importance ranking, presented as a bar chart based on the average absolute aggregated SurvSHAP value (Average |aggregated SurvSHAP(t)|), illustrating the cumulative contribution of the 16 features throughout the follow-up period. **(B)** Time trend plot of feature importance (based on the average absolute SurvSHAP value), demonstrating the dynamic evolution of each feature’s influence intensity over the follow-up period (0–1825 days).

Analysis of the mean absolute SHAP values (**Figure 13A**) revealed that ACCI ranked first in the overall contribution ranking with a mean absolute SHAP value of 0.060, followed by Surgery (0.049) and Mstage (0.048). TC (0.030) entered the top four. This ranking confirms that baseline health status (ACCI), treatment intervention (Surgery), and tumor metastasis status (Mstage) are features with the largest model-estimated contributions to predicted survival probability (24, 25). The time trend plot of feature importance (**Figure 13B**) illustrates the temporal variation of the mean absolute SurvSHAP values for each feature, revealing time-dependent patterns in the model’s feature attribution.

**Figure 14:**
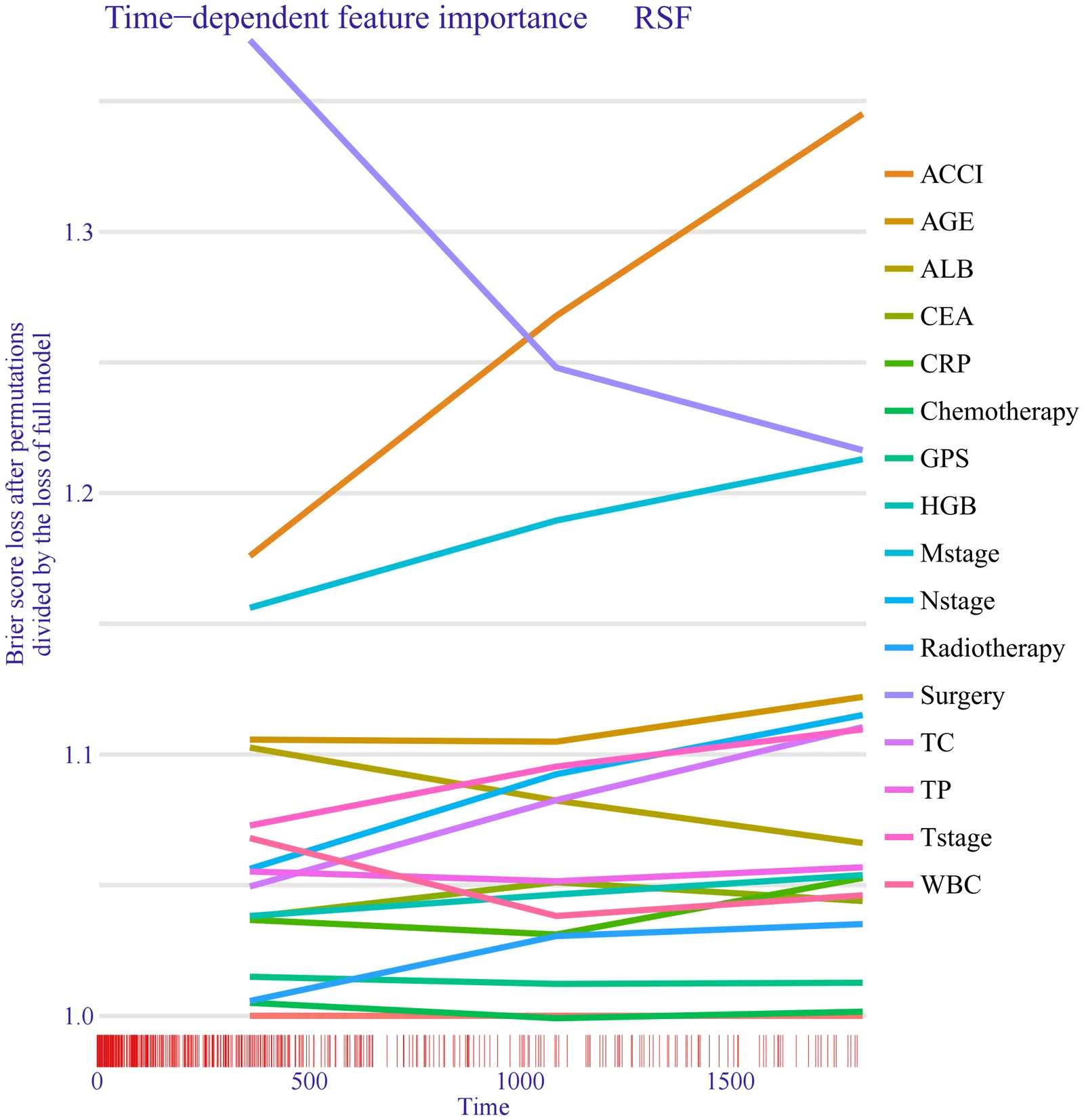
Time-dependent feature importance curves of the RSF model (based on permutation significance); the Y-axis shows the post-permutation brier score loss ratio relative to the full model, with higher values indicating greater feature importance; the red baseline indicates the distribution density over follow-up time.

The time-dependent average absolute value curve of SHAP (**Figure 14**) demonstrates that the model-estimated importance of ACCI increases continuously over time, peaking at 1500 days, reflecting the cumulative detrimental effects of comorbidities on long-term survival. The model-estimated importance of Surgery is higher in the early stage (within 500 days) and gradually declines in the middle stage, suggesting that the short-term benefits of surgical intervention are more pronounced. The model-estimated importance of Mstage is highest in the early stage (within 500 days) and then declines slowly, indicating that the impact of metastatic status on survival persists throughout the entire course but diminishes over time. The model-estimated importance of TC steadily increases over time, entering the top three at 1500 days, suggesting that the protective role of metabolic indicators on long-term survival becomes increasingly prominent. These dynamic changes are highly consistent with clinical cognition and provide data support for prognostic priorities at different follow-up stages (24, 25).

#### 3.5.2 Impact of characteristics on survival function

**Figure 15:**
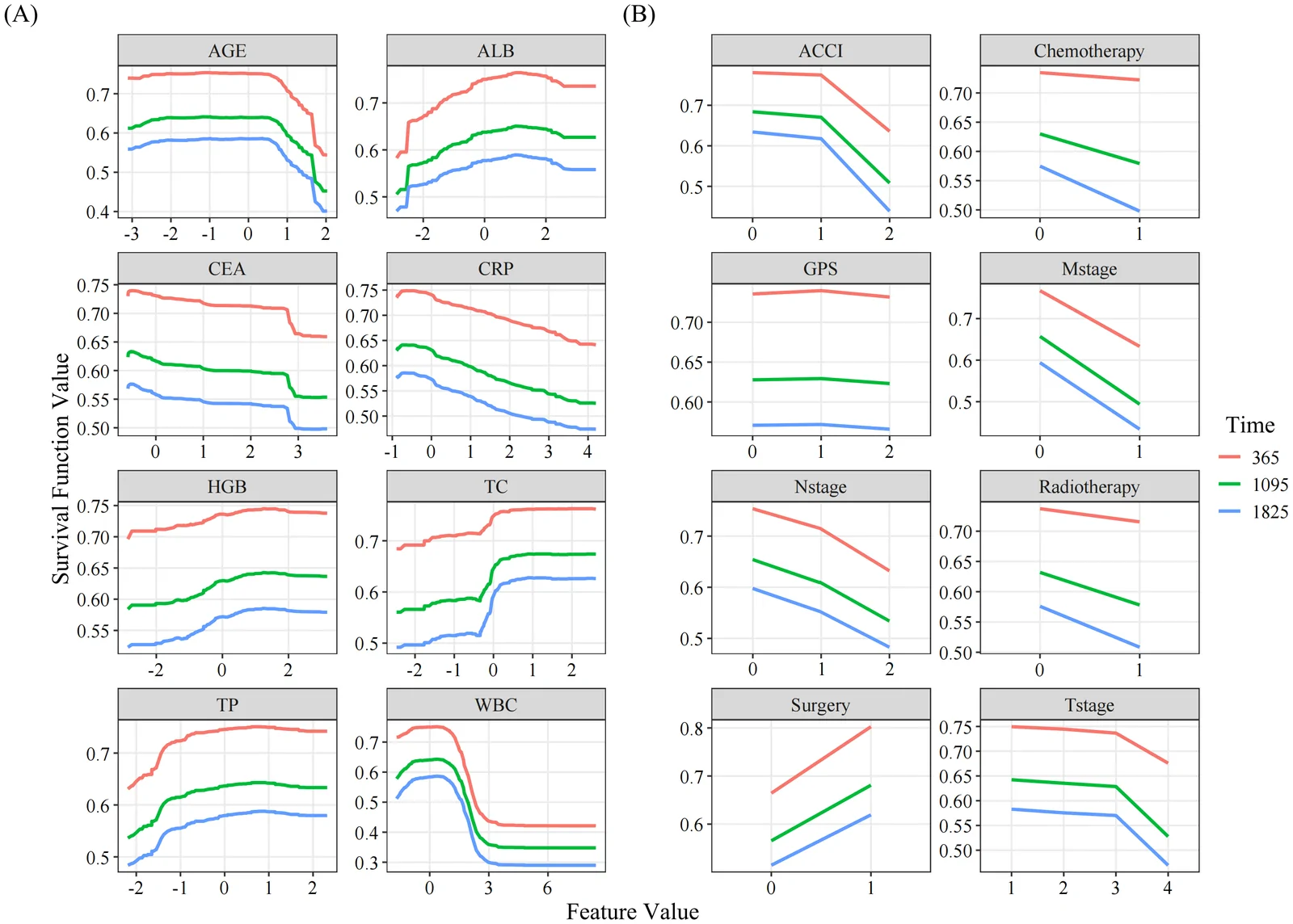
Partial dependence plot (PDP) analysis of the RSF model, demonstrating the marginal effects of 16 core predictive features on survival function values while controlling for other variables. The X-axis represents feature values (standardized for continuous variables, categorical variables at category levels), and the Y-axis shows survival function values (0–1). The red line (t = 365 days), green line (t = 1095 days), and blue line (t = 1825 days) correspond to 1-year, 3-year, and 5-year survival predictions, respectively. **(A)** Partial dependence curve for continuous features. **(B)** Partial dependence plot for categorical features.

Partial dependence plots (PDP) demonstrate the relationship between characteristic values and survival function. In continuous characteristics (**Figure 15A**), higher ALB and HGB levels were associated with higher model-predicted survival probabilities, consistent with established evidence linking nutritional status to CRC outcomes. Elevated CRP and CEA levels are associated with lower survival function values, suggesting that increased inflammatory responses and tumor markers are linked to poor prognosis. Higher AGE corresponds to lower survival function values, consistent with the negative impact of age on survival (24, 25). In categorical characteristics (**Figure 15B**), the survival curve for surgery (Surgery=1) was significantly higher than that for non-surgery (Surgery=0), consistent with established evidence for surgical benefit in CRC; however, this model association does not establish a causal therapeutic effect. The survival curve for distant metastasis (Mstage = 1) was markedly lower than that for no metastasis (Mstage = 0), consistent with the known adverse association between metastatic disease and survival. The survival curve for the comorbidity index (ACCI = 2) was lower than that for ACCI = 0/1, indicating that more severe comorbidities correlate with poorer survival prognosis (24, 25).

**Figure 16:**
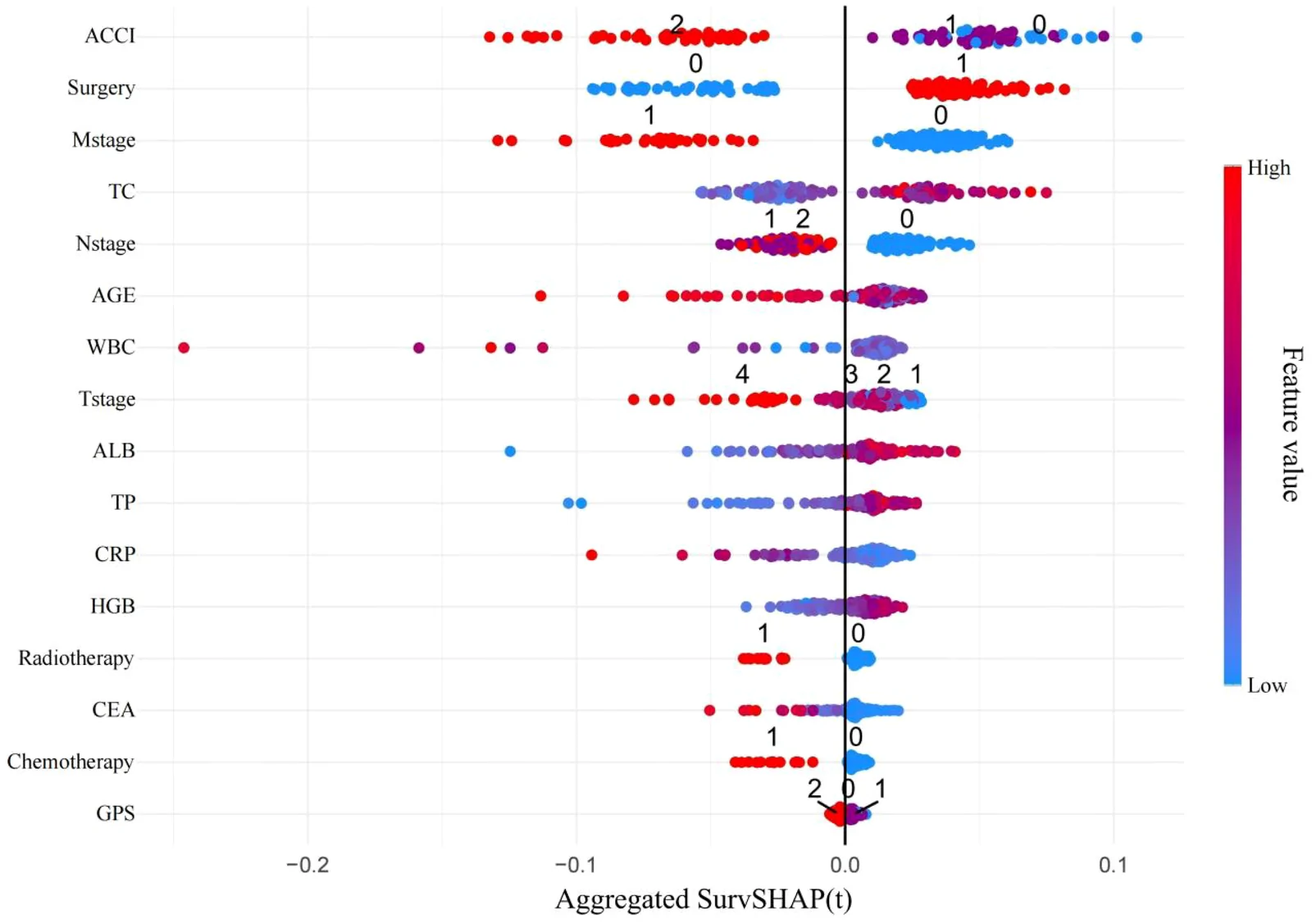
SHAP beeswarm plot based on the RSF model, illustrating the distribution of the impact of 16 key features on survival prediction in colorectal cancer patients. The vertical axis shows features ranked descendingly by importance (average absolute SurvSHAP values); the horizontal axis displays aggregated SurvSHAP(t) values, indicating each feature’s contribution to survival probability (positive values indicate increased survival probability, negative values indicate decreased survival probability). Point colors represent feature values (red for high values, blue for low values).

The SHAP dendrogram revealed specific association patterns between feature values and survival probabilities (**Figure 16**). Among categorical features, higher ACCI scores were associated with more negative SHAP values and lower survival probabilities; individuals with ACCI = 2 were almost entirely concentrated in the negative region. The SHAP value for Surgery=1 was significantly higher than 0, consistent with established evidence for surgical benefit in CRC; however, this model association does not establish a causal therapeutic effect. The SHAP value for Mstage=1 was negative, while Mstage=0 was positive, indicating a clear survival advantage in individuals without metastasis (24, 25). For continuous features, higher AGE was associated with lower SHAP values, showing a linear negative correlation. Higher TC was associated with higher SHAP values, demonstrating a linear positive correlation, suggesting that a favorable metabolic state may improve long-term survival. Higher ALB and HGB levels were associated with higher SHAP values, further confirming the protective role of nutritional status. Higher CRP levels were associated with lower SHAP values, highlighting inflammatory markers as potential risk stratification variables warranting investigation in interventional studies, such as controlling inflammation and improving nutrition (24, 25).

#### 3.5.3 Individual-level feature contribution (SHAP analysis)

The SurvSHAP(t) curve demonstrates the time-dependent contribution of each feature to individual survival probability (**Supplementary Figure 3**). The model-estimated contribution of ACCI shows a continuous decline over time, reaching its minimum at 1500 days, indicating that the detrimental effects of comorbidities on survival accumulate over time, with more pronounced long-term impacts. The model-estimated contribution of AGE gradually increases over time, suggesting that the negative effect of age on survival intensifies over time, with elderly patients exhibiting higher long-term survival risks. The model-estimated contribution of Surgery is relatively high in the early stage (within 500 days) and gradually declines thereafter, validating the more prominent short-term benefits of surgical intervention. The model-estimated contribution of TC steadily rises over time, ranking among the top three by 1500 days, indicating that the protective effect of metabolic markers on long-term survival becomes increasingly evident. These dynamic changes reveal time-heterogeneity in the impact of features on survival, providing a basis for prioritizing prognostic features at different follow-up stages (24, 25).

The SHAP force-directed graph (**Supplementary Figure 4**) illustrates the characteristic contributions of individuals at specific time points: ACCI = 2 was the most significant negative across all time points (SHAP value ≈-0.05), indicating a significantly increased survival risk in individuals with severe comorbidities. Mstage=1 had a SHAP value of ≈-0.04, demonstrating a detrimental effect of metastatic status on survival throughout the study period. Nstage=1 and Tstage=4 exhibited SHAP values of ≈-0.03, indicating that tumor progression was a critical prognostic risk factor. Low ALB (-1.08) and low HGB (-0.79) suggested reduced survival probabilities in individuals with abnormal nutritional biomarkers. Surgery=1 showed the largest positive SHAP value (≈+0.1) at all time points, consistent with higher model-predicted survival probability for patients receiving surgical treatment. Low CEA (-0.54) and normal WBC (0.14) indicated higher survival probabilities in individuals with normal tumor and inflammatory biomarkers. These results quantified the specific impact intensity of characteristics on individual survival, providing clear targets for clinical intervention (24, 25).

### 3.6 Web application

To enhance the clinical applicability of the model, the research team has encapsulated the optimized RSF model into an interactive web application (https://soochowf2yzxy.shinyapps.io/rsfmodel/) using R’s Shiny Web application framework. Users can predict the 1-year, 3-year, and 5-year survival probabilities of CRC patients by simply entering the patient’s baseline characteristics (i.e., the actual values of the 16 key features screened by the model, with GPS feature values automatically calculated). This provides clinicians with a convenient decision-support tool (28).

## 4 Discussion

### 4.1 Interpretational boundary statement

Throughout this manuscript, SHAP (SHapley Additive exPlanations) and PDP (Partial Dependence Plot) analyses are employed to explain how the RSF model attributes predictions to individual features. These explanations describe model behavior and do not establish causal relationships, therapeutic benefit, or clinical effectiveness. Feature importance rankings indicate which variables the model utilizes for prediction, not necessarily which variables causally influence patient outcomes. Treatment variables (e.g., Surgery) are included as predictors to approximate known prognostic associations; however, their inclusion does not permit causal inference about treatment efficacy from this retrospective observational design. All clinical implications discussed herein should be considered hypothetical pending prospective validation.

CRC is one of the most prevalent and lethal malignancies worldwide, and its prognosis prediction is crucial for guiding clinical treatment and improving patient survival (29). The traditional TNM staging system has limitations in assessing patient prognosis, such as significant differences in prognosis among patients with the same TNM stage. This has prompted researchers to explore more accurate prognostic prediction methods, where the integration of multidimensional biomarkers and machine learning techniques has become an important direction.

The stepwise incremental-value analysis provides a structured framework for understanding how distinct information domains contribute to prognostic refinement beyond conventional TNM staging. Notably, the largest single-domain increment derived from demographics and comorbidity (Model 1: ΔAUROC +0.077), followed by treatment modalities (Model 2: ΔAUROC +0.042 vs. Model 1). This pattern aligns with established evidence that host factors and therapeutic interventions are major determinants of CRC survival, yet these dimensions are absent from the AJCC anatomical staging system. Model 3 demonstrates that laboratory markers added to TNM alone provide modest incremental value (+0.032 vs. Model 0), but when demographics/comorbidity and treatment are already included (Model 2), laboratory markers contribute redundantly (−0.087 vs. Model 2), suggesting their prognostic information is largely captured by the more parsimonious clinical variables. This performance degradation may stem in part from collinearity between laboratory indicators and surgery/chemotherapy status after treatment variables have been incorporated, or from the model complexity of the 10 features in the external validation set of 561 cases being overfitting in nested comparison, thus highlighting the need for RSF to capture nonlinear interactions in the full 16-feature ensemble. But substantial contribution in the integrated full model (Model 4: ΔAUROC +0.122 vs. Model 3) suggests context-dependent synergy: inflammatory and nutritional markers may modulate prognosis primarily through interactions with comorbidity burden and treatment response rather than as independent linear predictors. This nonlinear dependency is consistent with the RSF algorithm’s capacity to capture feature interactions, which linear models may fail to exploit. We caution that the treatment variable increments in this retrospective design do not establish causal therapeutic benefit. The observed associations between surgical intervention and improved model predictions likely reflect, in part, selection of operable patients with favorable disease characteristics and performance status. The sensitivity analysis excluding treatment variables (RSF No Treat: AUROC 0.814) confirms a robust prognostic signal from non-treatment features, supporting potential application in pretreatment risk stratification, though prospective validation is essential.

The RSF model showed the highest comprehensive score among the nine compared algorithms, achieving a comprehensive score of 1.000 on the external validation set, with an average AUROC of 0.818, an average AUPR of 0.543, and an average Brier score of 0.120. RSF showed statistically higher AUROC than DT and LGBM (DeLong test, both P < 0.01), and significant improvement over XGB (P < 0.05). However, the performance advantages over other models, such as EN (AUROC 0.771) and Lasso (AUROC 0.768), did not reach statistical significance, providing an additional approach for CRC prognostic risk stratification in hospitalized patients.

The outstanding performance of the RSF model is closely related to its inherent advantages in handling survival data. RSF can model nonlinear relationships and interactions among features, which may better approximate complex prognostic patterns than linear approaches and treatment response (25). For instance, the joint effects of ACCI and Surgery are complex associations that linear models may not capture without explicit interaction terms (25). Additionally, RSF demonstrates relatively stable performance across the two distinct cohorts evaluated, though generalization to broader populations requires further validation (21). Other research has also confirmed the application value of Random Forest in cancer prediction, such as its use in constructing predictive models for breast cancer patient survival (25).

In terms of feature selection, this study utilized the Boruta algorithm to screen out 16 core features, which encompass key dimensions such as tumor progression (e.g., Mstage), therapeutic interventions (e.g., Surgery), and patient baseline status (e.g., ACCI), broadly concordant with established prognostic factors in CRC (21). Feature importance analysis further revealed that ACCI, Mstage, and Surgery are features with the largest model-estimated contributions in this study population (30). The importance of inflammatory markers (e.g., CRP), nutritional markers (e.g., ALB, TC), immune markers (e.g., WBC), and tumor markers (e.g., CEA) remained relatively stable across the entire time frame, suggesting potential utility as components of risk stratification tools, pending validation (30). Notably, the importance of these features dynamically changes over time, reflecting the complexity and dynamism of prognostic factors. For instance, Surgery within the short term (365 days) is a key feature, ACCI becomes the dominant factor in the medium term (1095 days), and ACCI and Surgery jointly dominate in the long term (1825 days) (30). This temporal dynamism aligns with previous findings that comorbidities are independent risk factors for long-term CRC prognosis (30).

The innovations of this study are reflected in the following aspects: First, integration of multiple routinely available data domains distinguishes this model from single-feature studies. Unlike studies that rely solely on a single type of feature (e.g., genomic data or clinical pathological features) (31), this study integrated eight categories of features, including clinical pathology, inflammation, immunity, nutrition, and treatment, as well as composite indicators such as GPS (30). This enables the model to capture complex prognostic information from a more comprehensive perspective, thereby potentially enhancing prognostic resolution in similar patient populations. Second, in terms of model optimization and validation, this study not only conducted thorough internal validation but also utilized independent datasets from different hospitals for external validation, providing initial evidence for model performance across distinct clinical settings, though multicenter prospective validation is required before clinical implementation (31). Finally, in clinical translation, this study developed a Shiny web application based on the RSF model, which can predict patients’ 1-year, 3-year, and 5-year survival probabilities in real time, providing an exploratory tool for prognostic estimation; its impact on clinical decision-making requires prospective evaluation (30).

The RSF model developed in this study, based on routinely available clinical and laboratory variables, may have potential clinical utility for risk stratification in hospitalized CRC patients. It may enable clinicians to more accurately assess patients’ prognostic risks and achieve more refined risk stratification. By predicting 1-year, 3-year, and 5-year survival probabilities, physicians may use model outputs as one component of risk stratification for discussion of treatment and follow-up options (32). For instance, risk stratification may inform discussions about follow-up intensity; however, treatment decisions should be based on established clinical evidence and patient preferences rather than model predictions alone (32). Feature importance analysis reveals associations captured by the model, which should not be interpreted as causal effects. For example, the model assigns higher importance to surgery in short-term predictions, which is consistent with clinical observations. However, these associations do not establish causal therapeutic effects, and treatment decisions should be based on established clinical evidence and patient-specific factors rather than model feature importance alone. Whereas the impact of ACCI on long-term survival is consistent with evidence supporting comprehensive complication management in CRC care (30). Additionally, the visualized results may facilitate patient-clinician communication regarding prognostic estimates; impact on decision-making or adherence requires dedicated study.

The MIMIC and SUFAH cohorts differ substantially in disease severity, comorbidity burden, metastatic status, and treatment patterns. While this heterogeneity enables assessment of model performance stability across divergent clinical phenotypes across different clinical settings, it also limits direct generalizability. Specifically, the MIMIC cohort represents a higher-acuity, more medically complex patient population (higher ACCI, more advanced T/N/M stages, lower surgery rates), whereas the SUFAH cohort represents a predominantly surgical population with earlier-stage disease and lower comorbidity burden. These differences should be interpreted as a test of model transportability rather than simple external validation, as the two cohorts represent distinct clinical phenotypes of CRC. The model’s performance in the SUFAH cohort demonstrated numerical stability across the evaluated disease severity spectrum, but clinicians should consider the specific patient population when applying the model in practice.

First, the generalizability of this model is limited by the data sources (30). The MIMIC-IV database is an electronic health record (EHR) database derived from a tertiary care center (Beth Israel Deaconess Medical Center) and primarily includes hospitalized and critically ill patients, who represent a higher-risk subgroup of the CRC population. The external validation set was obtained from a single-center cohort (SUFAH) in China. Therefore, the model is primarily applicable to hospitalized CRC patients with moderate-to-high disease severity and should not be generalized to the general outpatient CRC population or asymptomatic screening cohorts. Future multicenter studies encompassing diverse clinical settings and disease severities are needed to assess the model’s transportability (33). Second, this study mainly focused on clinical pathology and laboratory indicators, failing to include molecular markers such as ctDNA methylation and microsatellite instability (MSI) (31). With the development of high-throughput sequencing technology, novel biomarkers such as genomics, transcriptomics, proteomics, and metabolomics have shown great potential in cancer prognosis prediction (31). Future studies could attempt to integrate these “omics” data into the model to explore additional prognostic patterns and evaluate whether prediction accuracy improves in specific subpopulations (31). Finally, although the RSF model showed favorable discrimination and calibration in the evaluated cohorts, its underlying mechanisms are relatively complex, and its feature-level interpretability requires specialized methods (e.g., SHAP) that are less immediately transparent than linear coefficient estimates (34). Although this study enhanced interpretability through SHAP and PDPs, the clinical interpretability of tree-based model outputs warrants dedicated usability research, and more intuitive clinical decision support interfaces should be developed in the future (35).

Future research directions may include: First, multi-omics integration, which involves combining genomic, transcriptomic, and ctDNA dynamic monitoring data with clinical data to explore whether a ‘clinical + molecular’ dual-dimensional approach enhances prognostic resolution (31). Second, multicenter validation by expanding the sample to multi-center cohorts in Asia, Europe, and other regions to evaluate the performance stability of the RSF model approach across different ethnic groups (33). Additionally, prospective clinical trials should be conducted to evaluate whether model-informed risk stratification influences postoperative follow-up planning or treatment discussion patterns in randomized settings, thereby assessing its potential utility as a component of clinical decision support in real-world settings. Finally, efforts should be made to explore the development of dynamic prognostic prediction models, such as time-dependent covariate-based dynamic prediction models, combined with real-time data update mechanisms to track whether dynamic updates improve prediction alignment with observed outcomes (35).

## 5 Conclusion

Based on retrospective cohort data from two distinct clinical settings, this study constructed and evaluated an RSF prognostic model integrating routinely available clinical, laboratory, inflammatory, nutritional, and treatment variables. The model demonstrated favorable discrimination (external validation AUROC = 0.818, 95% CI: 0.761-0.866) and calibration (Brier score = 0.120) in predicting 1-year, 3-year, and 5-year survival, with statistically significantly higher AUROC than TNM-only staging (DeLong test P < 0.001); whether this translates to improved clinical outcomes requires prospective evaluation. Among the nine compared algorithms, RSF showed relatively stable performance rankings across internal and external validation. Through SHAP explanatory analysis, the study revealed that ACCI, Mstage, and Surgery were the features with the largest model-estimated contributions, characterizing time-dependent patterns in model feature contributions: The model assigned the highest importance to Surgery for short-term (1-year) survival benefits, to ACCI for intermediate-term (3-year) prognosis, and to both ACCI and Surgery for long-term (5-year) predictions. Additionally, inflammatory markers (CRP), nutritional markers (ALB, TC), and tumor markers (CEA) also exhibited stable predictive value.

This model provides additional prognostic information beyond the traditional TNM staging system in the evaluated cohorts in assessing prognostic heterogeneity, providing an exploratory quantitative tool for risk stratification that may inform discussions of treatment and follow-up strategies; impact on survival outcomes requires prospective clinical trials with the goal of aligning treatment intensity with estimated risk; however, the model’s effect on treatment patterns or survival has not been established.

The limitations of this study include: external validation based on single-center China population data, and the MIMIC database containing a large number of severe cases, which may limit the model’s generalizability in the general CRC population; the exclusion of molecular markers such as ctDNA methylation and MSI. Future studies need to further validate the model’s efficacy in multi-center prospective cohorts and integrate multi-omics data to improve predictive accuracy.

## Data Availability

The MIMIC-IV 3.1 database is publicly available via PhysioNet (https://physionet.org/content/mimiciv/3.1/). Access requires completion of the CITI Program training and a signed data use agreement. The single-center SUFAH cohort data supporting this study are not publicly available due to patient privacy regulations and institutional policy but may be made available upon reasonable request to the corresponding author with appropriate institutional ethics approval and data use agreements. Requests to access these datasets should be directed to XY, yangxiaodong2366@suda.edu.cn.

## Supplementary material

Supplementary Figure 1 Landmark sensitivity analysis: treatment variable temporal ordering. 7−day landmark on SUFAH external validation cohort (n=538 after excluding early events). Pre−landmark variant: only treatments occurring within 7 days of admission are included. Minimal performance decrement (Delta−AUROC −0.003 at 1−year) confirms robustness to treatment variable temporal ordering. RSF no treat (no treatment variables): mean AUROC 0.814.

Supplementary Figure 2 Stepwise incremental-value analysis of the RSF model hierarchy. **(A)** Bar plot of mean AUROC across the five nested models on the external validation set, with error bars representing 95% confidence intervals. **(B)** Waterfall plot of ΔAUROC relative to the immediate predecessor model, annotated with DeLong test P values. **(C)** Corresponding surv.cindex trajectory. **(D)** Calibration performance (mean Brier score) across model levels. The dashed horizontal line in **(A, C)** indicates the Full RSF (Model 4) performance for reference.

Supplementary Figure 3 The SurvSHAP(t) time-dependent feature importance analysis based on the RSF model. This plot illustrates the dynamic trend of how each clinical feature contributes to survival prediction over time. The X-axis represents follow-up duration (days), and the Y-axis shows the SurvSHAP(t) value (feature importance score): positive values indicate increased mortality risk, while negative values indicate reduced mortality risk.

Supplementary Figure 4 SHAP force plot for the RSF model at specific time points, illustrating how baseline predictions (approximately 0.5) are combined through the contributions of various features to form final survival probability estimates at three follow-up time points: t = 365 days (1 year), t = 1095 days (3 years), and t = 1825 days (5 years). Red bars represent negative contributions (reducing survival probability), while blue bars indicate positive contributions (increasing survival probability); numerical labels denote the corresponding SurvSHAP values.
